# Covariant Relativistic Non-Equilibrium Thermodynamics of Multi-Component Systems †

**DOI:** 10.3390/e21111034

**Published:** 2019-10-24

**Authors:** Wolfgang Muschik

**Affiliations:** Institut für Theoretische Physik, Technische Universität Berlin, Hardenbergstr. 36, D-10623 Berlin, Germany; muschik@physik.tu-berlin.de

**Keywords:** general-covariant multi-component systems, entropy identity, entropy balance of a component of the mixture, entropy balance of the mixture, multi-temperature relaxation, equilibrium conditions: 4-temperature’s Killing relation, extended Belinfante/Rosenfeld procedure, 2-component plain-ghost mixture

## Abstract

Non-equilibrium and equilibrium thermodynamics of an interacting component in a relativistic multi-component system is discussed covariantly by exploiting an entropy identity. The special case of the corresponding free component is considered. Equilibrium conditions and especially the multi-component Killing relation of the 4-temperature are discussed. Two axioms characterize the mixture: additivity of the energy momentum tensors and additivity of the 4-entropies of the components generating those of the mixture. The resulting quantities of a single component and of the mixture as a whole, energy, energy flux, momentum flux, stress tensor, entropy, entropy flux, supply and production are derived. Finally, a general relativistic 2-component mixture is discussed with respect to their gravitation generating energy–momentum tensors.

## 1. Introduction

The treatment of multi-component systems is often restricted to transport phenomena in chemically reacting systems, which means the mixture consisting of different components is shortly described by 1-component quantities such as temperature, pressure and energy that are not retraced to the corresponding quantities of the several components of the multi-component system. That is the case in non-relativistic physics [1,2,3] as well as in relativistic physics [4,5,6,7,8]. In this paper, the single component as an interacting member of the mixture is investigated. Thus, each component of the mixture is equipped with its own temperature, pressure, energy and mass density, which all together generate the corresponding quantities of the mixture.

Considering a multi-component system, three items have to be distinguished: one component as a member of the multi-component system which interacts with all the other components of the system; the same component as a free 1-component system separated from the multi-component system; and the multi-component system itself as a mixture. which is composed of its components. Here, all three items are discussed in a covariant-relativistic framework. For finding out the entropy-flux, -supply, -production and -density, a special tool is used: the entropy identity which constrains the possibility of an arbitrary choice of these quantities [9,10,11,12]. Following J. Meixner and J.U. Keller that entropy in non-equilibrium cannot be defined unequivocally [13,14,15,16,17], the entropy identity is only an (well set up) ansatz for constructing a non-equilibrium entropy and further corresponding quantities. This fact in mind, a specific entropy and the corresponding Gibbs and Gibbs–Duhem equations are derived. The definition of the rest mass flux densities, of the energy and momentum balances and of the corresponding balances of the spin tensor are taken into account as constraints in the entropy identity by introducing fields of Lagrange multipliers. The physical dimensions of these factors allow determining their physical meaning.

Equilibrium is defined by equilibrium conditions, which are divided into basic ones given by vanishing entropy-flux, -supply and -production and supplementary ones such as vanishing diffusion flux, vanishing heat flux and zero rest mass production [11,12]. The Killing relation of the 4-temperature concerning equilibrium is shortly discussed. Constitutive equations are out of scope of this paper. (According to the material theory, the strict distinction between (basic) balance equations, which are valid for arbitrary materials and constitutive equations characterizing special materials is made. Introducing the constitutive equations into the balances result in a set of differential equations, which solve the problem (see Section 9).)

The paper is organized as follows: After this Introduction, the kinematics of a multi-component system is considered in the next two sections for introducing the mass flux and the diffusion flux densities. The energy–momentum tensor is decomposed into its (3+1)-split, and the entanglement of the energy and momentum balances are discussed, followed by non-equilibrium thermodynamics of an interacting component of the mixture and that of the corresponding free component. The equilibrium of both is considered. Thermodynamics of the mixture starts with three axioms: additivity of the mass flux densities, of the energy momentum tensors and of the 4-entropies of the components resulting in those of the mixture. Entropy, and entropy-flux, -supply and -production are found. The paper finishes discussing the gravitation generated by a special general-relativistic 2-component system: one component equipped with a symmetric energy–momentum tensor, a mass density and vanishing external force density, the other one with a skew-symmetric energy–momentum tensor, an external force density and vanishing mass density. A summary and an appendix are added.

## 2. Kinematics

### 2.1. The Components

We consider a multi-component system consisting of *Z* components. The component index ${}^{A}$ runs from 1 to *Z*. Each component has its own rest frame $B^{A}$ in which the rest mass density $\varrho^{A}$ is locally defined (more details in Appendix A.1) These remaining mass densities are relativistic invariants and therefore frame independent.

In general, the components have different 4-velocities, namely $u_{k}^{A},\;A=1,2,\ldots ,Z;k=1,\ldots ,4,$ which all are tensors of first order under Lorentz transformation. We now define the component mass flux density as a 4-tensor of first order and the component mass production term as a scalar

(1) $$N_{k}^{A}\;:=\;\varrho^{A}u_{k}^{A},\;N^{Ak}{}_{;k}\;=\;\Gamma^{A},\;N_{k}^{A}N^{Ak}\;=\;{\left(\varrho^{A}\right)}^{2}c^{2}\geq 0.$$

Here, Equation (1)${}_{2}$ is the mass balance equation of the ${}^{A}$-component. Consequently, we introduce the basic fields of the components

(2) $$\left\{\varrho^{A},\;u_{k}^{A}\right\},\;\varrho^{A}\;\geq \;0,\;u_{k}^{A}u^{Ak}\;=\;c^{2},\;\forall A$$

The mass production term has two reasons: an external one by mass supply and one internal one by chemical reactions

(3) $$\Gamma^{A}\;={\;}^{\left(ex\right)}\Gamma^{A}{+}^{\left(in\right)}\;\Gamma^{A}.$$

The external mass supply ${}^{\left(ex\right)}\Gamma^{A}$ depends on the environment of the system, whereas ${}^{\left(in\right)}\Gamma^{A}$ is determined by chemical reactions depending on the set of frame-independent stoichiometric equations, which are discussed in Appendix A.3.

### 2.2. The Mixture

As each component, the multi-component system also has a mass density ϱ and a 4-velocity $u_{k}$, which are determined by the partial quantities of the components. For deriving ϱ and $u_{k}$, we apply the nearly self-evident: 

■ Mixture Axiom: The balance equation of a mixture looks like the balance equation of a one-component system. ■ 

Especially here, the mixture axiom is postulated for the balance equations of mass, energy–momentum and entropy. According to the mixture axiom, the mass balance of the mixture looks according to (1)${}_{2}$

(4) $$N^{k}{}_{;k}\;=\;\Gamma ,\;\Gamma \;=\;0,$$

with vanishing total mass production, if the mass of the mixture is conserved (the mixture as a closed system).

Now, the question arises: Which quantities of the components of the mixture are additive? Obviously, neither the mass densities $\varrho^{A}$ nor the 4-velocities $u_{k}^{A}$ are additive quantities according to their definitions. Consequently, we demand in accordance with the mixture axiom that the mass flux densities are additive. (The sign $\overset{•}{=}$ stands for a setting and := for a definition which is a formal short form for an expression without any physical background. A setting is a definition induced by physics determining the axiomatic structure of a theory.) That is,

$$\begin{array}{c} \mathrm{Setting}I:\; \end{array}$$

(5) $$\begin{array}{c} \sum_{A}N_{k}^{A}\;\overset{•}{=}\;N_{k}\;\underset{↓}{\underset{︸}{=}}\;\varrho u_{k}\;\underset{↓}{\underset{︸}{=}}\;\sum_{A}\varrho^{A}u_{k}^{A}\;⟶\;u_{k}\;=\;\sum_{A}\frac{\varrho^{A}}{\varrho }u_{k}^{A}. \end{array}$$

(6) $$\begin{array}{c} \mathrm{mixture}\;\mathrm{axiom}\;{\left(1\right)}_{1}\; \end{array}$$

For the present, mass density ϱ and 4-velocity $u_{k}$ of the mixture are unknown. Of course, they depend on the basic fields of the components in Equation (2). Contraction with $u^{k}$ and use of Equation (5)${}_{2,3}$ results in

(7) $$\varrho \;=\;\frac{1}{c^{2}}\sum_{A}\varrho^{A}u_{k}^{A}u^{k}\;=\;\frac{1}{c^{2}}N_{k}u^{k}\;=\;\frac{1}{c^{2}}N_{k}\frac{1}{\varrho }N^{k}\;⟶\;\varrho \;=\;\pm \frac{1}{c}\sqrt{N_{k}N^{k}},$$

(the sign is determined below) or in more detail

(8) $$\varrho \;=\;\pm \frac{1}{c}\sqrt{\sum_{A,B}\varrho^{A}\varrho^{B}u_{k}^{A}u^{Bk}}.$$

The mass density ϱ and the 4-velocity $u_{k}$ of the mixture are expressed by those of the components according to Equations (8) and (5)${}_{4}$. According to Equation (5)${}_{4}$, the 4-velocity of the mixture is a weighted mean value of the 4-velocities of the components. For the mass density, we have according to Equation (7)${}_{1}$ also a with the Kluitenberg factor $f^{A}$ (>0 results from the representation of the 4-velocities in components) weighted mean value of the mass density components [18]

In Equation (7)${}_{1}$ appears the Kluitenberg factor [18]

$$\begin{array}{ccc} f^{A} & := & \frac{1}{c^{2}}u_{k}^{A}u^{k}\;=\;\frac{1}{c^{2}}\left(u_{\alpha }^{A}u^{\alpha }+u_{4}^{A}u^{4}\right)\;= \end{array}$$

(9) $$\begin{array}{ccc} & = & \frac{1}{c^{2}}\left\{\left(-v^{A}\cdot v+c^{2}\right){\left(1-v^{A}/c^{2}\right)}^{-1/2}{\left(1-v/c^{2}\right)}^{-1/2}\right\}\;>\;0\; \end{array}$$

(10) $$\begin{array}{ccc} \;⟶\;\varrho & = & \sum_{A}f^{A}\varrho^{A}\;=\;\sum_{A}f^{A}\left(u_{k}^{A},u^{k}\right)\varrho^{A}\;>\;0, \end{array}$$

resulting according to Equations (7)${}_{1}$ and (5)${}_{4}$ in the entanglement of ϱ and $u_{k}$, which are not independent of each other

(11) $$\varrho \;=\;R\left(\varrho^{A},u_{k}^{A},u_{k}\right),\;u_{k}\;=\;U_{k}\left(\varrho^{A},u_{k}^{A},\varrho \right).$$

A comparison of Equation (10) with Equation (7)${}_{4}$ results in

(12) $$N_{k}N^{k}\;>\;0\;⟶\;u_{k}u^{k}\;>\;0,$$

according to Equation (5)${}_{2}$, thus demonstrating that $u^{k}$ is really a 4-velocity.

According to Equations (5)${}_{1}$ and (1)${}_{2}$, we obtain the additivity of the mass production terms

(13) $$N^{k}{}_{;k}\;=\;\sum_{A}N^{Ak}{}_{;k}\;=\;\sum_{A}\Gamma^{A}\;=\;\Gamma \;⟶\;\sum_{A}{}^{ex}\Gamma^{A}\;={\;}^{ex}\Gamma ,\;\sum_{A}{}^{in}\Gamma^{A}\;=\;{}^{in}\Gamma .$$

The sign of ${}^{in}\Gamma$ depends on the considered reaction: it vanishes for chemical reactions and may be different for nuclear ones.

### 2.3. The Diffusion Flux

From Equations (5)${}_{3}$ and (9)${}_{2}$ follows

(14) $$0\;=\;\sum_{A}\varrho^{A}u_{k}^{A}-u_{k}\sum_{A}f^{A}\varrho^{A}\;=\;\sum_{A}\varrho^{A}\left(u_{k}^{A}-f^{A}u_{k}\right).$$

Introducing the diffusion flux density

(15) $$J_{k}^{A}\;:=\;\varrho^{A}\left(u_{k}^{A}-f^{A}u_{k}\right)\;=\;N_{k}^{A}-\varrho^{A}f^{A}u_{k}\;⟶\;\sum_{A}J_{k}^{A}\;=:\;J_{k}\;=\;0,$$

we obtain

(16) $$\begin{array}{ccc} J_{k}^{A}u^{k} & = & \varrho^{A}\left(u_{k}^{A}u^{k}-f^{A}c^{2}\right)\;=\;0, \end{array}$$

(17) $$\begin{array}{ccc} J_{k}^{A}u^{Ak} & = & c^{2}\varrho^{A}\left[1-{\left(f^{A}\right)}^{2}\right]\;=:\;c^{2}\varrho^{A}w^{A}\;=\;w^{A}N_{k}^{A}u^{Ak}, \end{array}$$

(18) $$\begin{array}{c} 1-w^{A}\;\geq \;0.\; \end{array}$$

By introducing the projectors

(19) $$h_{l}^{Am}\;:=\;\delta_{l}^{m}-\frac{1}{c^{2}}u^{Am}u_{l}^{A},\;h_{l}^{m}\;:=\;\delta_{l}^{m}-\frac{1}{c^{2}}u^{m}u_{l},$$

we obtain the following properties of the diffusion flux density:

(20) $$\begin{array}{ccc} J^{Am}h_{m}^{k} & = & J^{Ak}\;=\;N^{Am}h_{m}^{k} \end{array}$$

(21) $$\begin{array}{ccc} J^{Am}h_{m}^{Ak} & = & \varrho^{A}f^{A}\left(f^{A}u^{Ak}-u^{k}\right) \end{array}$$

(22) $$\begin{array}{ccc} J^{Ak} & = & J^{Am}h_{m}^{Ak}+\varrho^{A}w^{A}u^{Ak}\;=\;J^{Am}h_{m}^{Ak}+w^{A}N^{Ak} \end{array}$$

(23) $$\begin{array}{ccc} J^{Ak}{}_{;k} & = & {\left(J^{Am}h_{m}^{Ak}\right)}_{;k}+\left(\varrho^{A}w^{A}\right){}_{;k}u^{Ak}+\varrho^{A}w^{A}u^{Ak}{}_{;k}. \end{array}$$

According to Equation (20)${}_{2}$, the diffusion flux density is that part of the mass flux density which is perpendicular to the 4-velocity of the mixture. The diffusion flux density vanishes in 1-component systems ($u_{k}^{A}\equiv u_{k}$) according to $f^{A}=f=1$ and Equation (15)${}_{1}$.

## 3. The Energy–Momentum Tensor

### 3.1. Free and Interacting Components

The energy–momentum tensor $T^{Akl}$ of the ${}^{A}$-component consists of two parts

(24) $$T^{Akl}\;=\;\overset{{}_{0}}{T}\;{}^{Akl}+\sum_{B}W_{B}^{Akl},\;W_{B}^{Bkl}\;=\;0.$$

Here, $\overset{{}_{0}}{T}\;{}^{Akl}$ is the energy–momentum tensor of the free ${}^{A}$-component, which is the case if there are no interactions between the ${}^{A}$-component and the other ones. $W_{B}^{Akl}$ describes the interaction between the ${}^{B}$-component and the ${}^{A}$-component. The interaction between the external environment and the ${}^{A}$-component is given by the external force density $k^{Al}$, which appears in the energy–momentum balance equation

(25) $$T^{Akl}{}_{;k}\;=\;k^{Al}\;=\;\Omega^{Al}+\frac{1}{c^{2}}u^{Al}u_{m}^{A}k^{Am},\;\Omega^{Al}u_{l}^{A}\;=\;0,$$

and in the balance equations of

(26) $$\begin{array}{ccc} \mathrm{energy}:\;u_{l}^{A}T^{Akl}{}_{;k} & = & u_{l}^{A}k^{Al}\;=:\;\Omega^{A}, \end{array}$$

(27) $$\begin{array}{ccc} \mathrm{and}\;\mathrm{momentum}:\;h_{l}^{Am}T^{Akl}{}_{;k} & = & h_{l}^{Am}k^{Al}\;=:\;\Omega^{Am}. \end{array}$$

Consequently, the interaction of the ${}^{A}$-component with the other components of the mixture modifies the energy–momentum tensor of the free ${}^{A}$-component. Additionally, its interaction with the environment shows up in the source of the energy–momentum balance. According to its definition, $T^{Akl}$ is the energy–momentum tensor of the “${}^{A}$-component in the mixture”.

### 3.2. (3+1)-Split

The (3+1)-split of the in general non-symmetric energy–momentum tensor of the ${}^{A}$-component is

(28) $$T^{Akl}\;=\;\frac{1}{c^{2}}e^{A}u^{Ak}u^{Al}+u^{Ak}p^{Al}+\frac{1}{c^{2}}q^{Ak}u^{Al}+t^{Akl}.$$

This energy–momentum tensor of the material theory is not induced by a Lagrangian or a variational problem. It presents a quantity on its own. An example is the energy–momentum tensor which is needed for describing liquid crystals or polar fluids: it is non-symmetric because the stress tensor $t^{kl}$ of these materials is non-symmetric and a Lagrangian with respect to the equations of motion—here the balance equations of the basic fields—does not exist. This is the reason Equation (28) is used in this paper.

The (3+1)-components of the energy–momentum tensor in Equation (28) are (the (3+1)-split is made by taking the physical meaning of Equations (29) and (30) into account, see Equation (36) to Equation (39))

(29) $$\begin{array}{c} e^{A}\;:=\;\frac{1}{c^{2}}T^{Ajm}u_{j}^{A}u_{m}^{A},\;p^{Al}\;:=\;\frac{1}{c^{2}}h_{m}^{Al}T^{Ajm}u_{j}^{A}, \end{array}$$

(30) $$\begin{array}{c} q^{Ak}\;:=\;h_{j}^{Ak}T^{Ajm}u_{m}^{A},\;t^{Akl}\;:=\;h_{j}^{Ak}T^{Ajm}h_{m}^{Al}, \end{array}$$

(31) $$\begin{array}{c} q^{Ak}u_{k}^{A}=0,\;p^{Al}u_{l}^{A}=0,\;t^{Akl}u_{k}^{A}=0,\;t^{Akl}u_{l}^{A}=0. \end{array}$$

The (3+1)-split of tensors is a usual tool in relativistic continuum physics. The (3+1)-components—generated by the split—have physical significance which originally is hidden in the unsplit tensors. Thus, we generate by (3+1)-splitting the following covariant quantities of the ${}^{A}$-component: the energy density $e^{A}$, the momentum flux density $p^{Al}$, the energy flux density $q^{Ak}$, the stress tensor $t^{Akl}$.

The symmetric part of the energy–momentum tensor in Equation (28) is

(32) $$T^{A(kl)}\;=\;\frac{1}{c^{2}}e^{A}u^{Ak}u^{Al}+\frac{1}{2c}u^{Ak}\left(cp^{Al}+\frac{1}{c}q^{Al}\right)+\frac{1}{2c}\left(cp^{Ak}+\frac{1}{c}q^{Ak}\right)u^{Al}+t^{A(kl)},$$

and its anti-symmetric part is

(33) $$T^{A[kl]}\;=\;\frac{1}{2c}u^{Ak}\left(cp^{Al}-\frac{1}{c}q^{Al}\right)-\frac{1}{2c}\left(cp^{Ak}-\frac{1}{c}q^{Ak}\right)u^{Al}+t^{A[kl]}.$$

The stress tensor is composed of the pressure $p^{A}>0,\forall A$, and the viscous tensor $\pi^{Akl}$

(34) $$t^{Akl}\;=\;-p^{A}h^{Akl}+\pi^{Akl},\;t_{k}^{Ak}\;=\;-3p^{A}.$$

For interpreting the (3+1)-components, we now consider their physical dimensions. According to Equations (19) and (9)${}_{1}$, we have (the bracket [⊠] signifies the physical dimension of ⊠)

(35) $$\left[h_{m}^{Al}\right]\;=\;1,\;\left[f^{A}\right]\;=\;1.$$

By taking Equations (34), (35)${}_{1}$ and (28) into account, we obtain

(36) $$\begin{array}{ccc} \left[t^{Akl}\right]\;=\;\left[p^{A}\right]\;=\;\left[\pi^{Akl}\right]\;=\;\left[e^{A}\right]\;=\;\left[q^{Ak}\right]\frac{s}{m} & = & \left[p^{Al}\right]\frac{m}{s},\\ \mathrm{pressure}\;=\;\left[p^{A}\right]\;=\;\frac{N}{m^{2}}\;=\;\frac{Nm}{m^{3}} & = & \mathrm{energy}\;\mathrm{density}\;=\; \end{array}$$

(37) $$\begin{array}{ccc} =\;\frac{kg\;m}{s^{2}}\frac{1}{m^{2}}\;=\;kg\frac{m}{s}\frac{1}{m^{3}}\frac{m}{s} & = & \mathrm{momentum}\;\mathrm{flux}\;\mathrm{density}, \end{array}$$

(38) $$\begin{array}{ccc} \left[q^{Ak}\right]\;=\;\left[e^{A}\right]\frac{m}{s}\;=\;\frac{Nm}{m^{3}}\frac{m}{s} & = & \mathrm{energy}\;\mathrm{flux}\;\mathrm{density}, \end{array}$$

(39) $$\begin{array}{ccc} \left[p^{Al}\right]\;=\;kg\frac{m}{s}\frac{1}{m^{3}} & = & \mathrm{momentum}\;\mathrm{density}. \end{array}$$

The (3+1)-split in Equation (28) of the energy–momentum tensor can be written in a more compact form

(40) $$\begin{array}{c} T^{Akl}\;=\;\frac{1}{c^{2}}Q^{Ak}u^{Al}+\tau^{Akl},\; \end{array}$$

(41) $$\begin{array}{c} u_{l}T^{Akl}\;=:\;Q^{Ak}\;=\;e^{A}u^{Ak}+q^{Ak},\;h_{l}^{m}T^{Akl}\;=:\;\tau^{Akm}\;=\;u^{Ak}p^{Am}+t^{Akm}. \end{array}$$

The energy–momentum tensor in Equation (40) is that of the ${}^{A}$-component in the mixture, which means—as discussed in Section 3.1—the ${}^{A}$-component is not a free system and the (3+1)-split-components $e^{A},q^{Ak},p^{Al}$ and $t^{Akl}$ include the internal interaction of the ${}^{A}$-component with all the other ones.

### 3.3. Additivity

We now consider the equivalent-system composed of the *Z* components: that is the mixture which consists of these *Z* interacting components. Because this interaction is already taken into account by the (3+1)-split-components, the energy–momentum tensors of the components are additive without additional interaction terms. Consequently, the energy–momentum tensor $T^{kl}$ of the mixture is

(42) $$\begin{array}{c} \mathrm{Setting}\mathrm{II}:\;\\ T^{kl}\;:=\;\frac{1}{c^{2}}Q^{k}u^{l}+\tau^{kl}\overset{•}{=}\;\sum_{A}T^{Akl}\;=\;\sum_{A}\left(\frac{1}{c^{2}}Q^{Ak}u^{Al}+\tau^{Akl}\right). \end{array}$$

Multiplication with $u_{l}$ results by use of Equations (9)${}_{1}$ and (41)${}_{2}$ in

(43) $$Q^{k}\;=\;\sum_{A}\left(Q^{Ak}f^{A}+\tau^{Akl}u_{l}\right),$$

and by multiplication with $h_{l}^{m}$, Equation (42) results in

(44) $$\begin{array}{ccc} \tau^{km} & = & \sum_{A}\left(Q^{Ak}g^{Am}+\tau^{Akl}h_{l}^{m}\right), \end{array}$$

(45) $$\begin{array}{ccc} g^{Am} & := & \frac{1}{c^{2}}u^{Al}h_{l}^{m}\;=\;\frac{1}{c^{2}}\left(u^{Am}-f^{A}u^{m}\right)\;=\;\frac{1}{c^{2}\varrho^{A}}J^{Am}. \end{array}$$

For a 1-component system ($u_{k}^{A}\equiv u_{k}$), we obtain according to Equation (45) $g^{Am}=g^{m}=0$ taking $f^{A}=f=1$ into account.

### 3.4. (3+1)-Components of the Mixture

Starting with Equation (41), we obtain

(46) $$\begin{array}{ccc} Q^{Ak}u_{k}^{A}\;=\;c^{2}e^{A}, & \; & Q^{Ak}h_{k}^{Am}\;=\;q^{Am}, \end{array}$$

(47) $$\begin{array}{ccc} \tau^{Akm}u_{k}^{A}\;=\;c^{2}p^{Am}, & \; & \tau^{Akm}h_{k}^{Aj}\;=\;t^{Ajm}. \end{array}$$

According to Equations (40) and (42)${}_{1}$, these relations are analogous to those of the mixture. Consequently, from Equation (43) follows

(48) $$Q^{k}u_{k}\;=:\;c^{2}e\;=\;\sum_{A}\left(Q^{Ak}f^{A}u_{k}+\tau^{Akl}u_{l}u_{k}\right),$$

and with Equation (46)${}_{1}$ resulting in the energy density of the mixture

(49) $$c^{2}e\;=\;\sum_{A}\left(c^{2}e^{A}{\left(f^{A}\right)}^{2}+q^{Ak}f^{A}u_{k}+c^{2}p^{Al}f^{A}u_{l}+t^{Akl}u_{l}u_{k}\right).$$

From Equation (43) follows

(50) $$Q^{k}h_{k}^{m}\;=:\;q^{m}\;=\;\sum_{A}\left(f^{A}Q^{Ak}h_{k}^{m}+h_{k}^{m}\tau^{Akl}u_{l}\right),$$

and with Equation (46)${}_{2}$ resulting in the energy flux density of the mixture

(51) $$q^{m}\;=\;\sum_{A}\left(c^{2}e^{A}f^{A}g^{Am}+q^{Ak}f^{A}h_{k}^{m}+c^{2}p^{Al}g^{Am}u_{l}+t^{Akl}h_{k}^{m}u_{l}\right).$$

From Equation (44) follows

(52) $$\tau^{km}u_{k}\;=:\;c^{2}p^{m}\;=\;\sum_{A}\left(Q^{Ak}u_{k}g^{Am}+\tau^{Akl}h_{l}^{m}u_{k}\right),$$

and with Equation (47)${}_{1}$ resulting in the momentum density of the mixture

(53) $$c^{2}p^{m}\;=\;\sum_{A}\left(c^{2}e^{A}f^{A}g^{Am}+q^{Ak}u_{k}g^{Am}+c^{2}p^{Al}f^{A}h_{l}^{m}+t^{Akl}h_{l}^{m}u_{k}\right).$$

From Equation (47)${}_{2}$ follows finally

(54) $$\tau^{km}h_{k}^{j}\;=:\;t^{jm}\;=\;\sum_{A}\left(Q^{Ak}g^{Am}h_{k}^{j}+\tau^{Akl}h_{l}^{m}h_{k}^{j}\right)$$

which by taking Equation (41) into account results in the stress tensor and the pressure of the mixture

(55) $$\begin{array}{ccc} t^{jm} & = & \sum_{A}\left(c^{2}e^{A}g^{Aj}g^{Am}+q^{Ak}h_{k}^{j}g^{Am}+c^{2}p^{Al}g^{Aj}h_{l}^{m}+t^{Akl}h_{k}^{j}h_{l}^{m}\right),\\ p & = & -\frac{1}{3}t^{jm}h_{jm}\;=\;-\frac{1}{3}\sum_{A}\left(Q^{Ak}g^{Am}h_{k}^{j}+\tau^{Akl}h_{l}^{m}h_{k}^{j}\right)h_{jm}\;=\\ & & \;=\;-\frac{1}{3}\sum_{A}\left(\frac{1}{c^{2}}Q^{Ak}u^{Ap}h_{kp}+\tau^{Akl}h_{kl}\right)\;= \end{array}$$

(56) $$\begin{array}{ccc} & & \;=\;-\frac{1}{3}\sum_{A}\left(\frac{1}{c^{2}}\left(e^{A}u^{Ak}+q^{Ak}\right)u^{Ap}h_{kp}+\left(u^{Ak}p^{Al}+t^{Akl}\right)h_{kl}\right). \end{array}$$

The additivity of the energy–momentum tensors in Equation (42) results in Equations (49), (51), (53) and (55), relations which express the (3+1)-components of the energy–momentum tensor of the mixture as those of the components and their velocities

(57) $$\begin{array}{ccc} \left\{e,q^{k},p^{k},t^{kl}\right\} & = & F\left(e^{A},q^{Ak},p^{Ak},t^{Akl},u^{Ak},\varrho \left(\varrho^{A},u_{k}^{A},u^{k}\right),u^{k}\left(\varrho^{A},u_{k}^{A},\varrho \right)\right), \end{array}$$

(58) $$\begin{array}{ccc} T^{kl} & = & \frac{1}{c^{2}}eu^{k}u^{l}+u^{k}p^{l}+\frac{1}{c^{2}}q^{k}u^{l}+t^{kl},\;t^{kl}\;=\;-ph^{kl}{+}^{⋄}\;\;\pi^{kl}. \end{array}$$

The 4-velocity $u^{k}$ is given by Equation (5)${}_{4}$.

The influence of the additivity of the energy–momentum tensors on the balance equations of energy and momentum is investigated in the next section.

## 4. Entanglement of Energy and Momentum Balances

If the energy-momentum tensors of the ${}^{A}$-component and of the mixture are $T^{Akl}$ and $T^{kl}$, the energy and momentum balances are according to the mixture axiom by use of Equations (26) and (27)

(59) $$\begin{array}{ccc} \mathrm{energy}:\;u_{l}^{A}T^{Akl}{}_{;k}\;=\;\Omega^{A}, & \; & u_{l}T^{kl}{}_{;k}\;=\;\Omega , \end{array}$$

(60) $$\begin{array}{ccc} \mathrm{momentum}:\;h_{l}^{Am}T^{Akl}{}_{;k}\;=\;\Omega^{Am}, & \; & h_{l}^{m}T^{kl}{}_{;k}\;=\;\Omega^{m}. \end{array}$$

The balances in Equations (59)${}_{3}$ and (60)${}_{3}$ follow from Equations (26) and (27) by the mixture axiom. Here, $\Omega^{A}$ and Ω are the energy supplies, and $\Omega^{Am}$ and $\Omega^{m}$ the momentum supplies of the ${}^{A}$-component and of the mixture.

The (3+1)-split of the divergence of the energy–momentum tensor of the ${}^{A}$-component results by use of Equation (19)${}_{1}$ in

(61) $$\delta_{l}^{m}T^{Akl}{}_{;k}\;=\;T^{Akm}{}_{;k}\;=\;h_{l}^{Am}T^{Akl}{}_{;k}+\frac{1}{c^{2}}u^{Am}u_{l}^{A}T^{Akl}{}_{;k}.$$

If the component index ${}^{A}$ is cancelled in Equation (61), we obtain the decomposition of the divergence of the energy–momentum tensor of the mixture. Taking Equations (59) and (60) into account, these divergences can be written as

(62) $$T^{Akm}{}_{;k}\;=\;\Omega^{Am}+\frac{1}{c^{2}}u^{Am}\Omega^{A},\;T^{km}{}_{;k}\;=\;\Omega^{m}+\frac{1}{c^{2}}u^{m}\Omega .$$

The additivity of the energy–momentum tensors in Equation (42) results in the additivity of the force densities (this is a strong argument supporting the validity of Setting II in Equation (42))

(63) $$k^{m}\;=\;\Omega^{m}+\frac{1}{c^{2}}u^{m}\Omega \;=\;\sum_{A}\left(\Omega^{Am}+\frac{1}{c^{2}}u^{Am}\Omega^{A}\right)\;=\;\sum_{A}k^{Am}.$$

Taking Equations (26)${}_{2}$ and (27)${}_{2}$ into account, we obtain by multiplication of Equation (63) with $u_{m}$ and $h_{m}^{p}$, respectively,

(64) $$\Omega \;=\;\sum_{A}\left(\Omega^{Am}u_{m}+f^{A}\Omega^{A}\right),\;\Omega^{p}\;=\;\sum_{A}\left(\Omega^{Am}h_{m}^{p}+g^{Ap}\Omega^{A}\right).$$

Inserting Equations (26) and (27), we obtain in more detail

(65) $$\begin{array}{c} u_{l}T^{kl}{}_{;k}\;=\;\sum_{A}\left\{\left(h_{l}^{Am}u_{m}+f^{A}u_{l}^{A}\right)T^{Akl}{}_{;k}\right\}, \end{array}$$

(66) $$\begin{array}{c} h_{l}^{p}T^{kl}{}_{;k}\;=\;\sum_{A}\left\{\left(h_{l}^{Am}h_{m}^{p}+g^{Ap}u_{l}^{A}\right)T^{Akl}{}_{;k}\right\}. \end{array}$$

As Equation (64) indicates, the additivity of the energy–momentum tensors causes that the supplies of energy and momentum are entangled, expressed by the inequalities

(67) $$\sum_{A}f^{A}\Omega^{A}\;\neq \;\Omega ,\;\sum_{A}\Omega^{Am}h_{m}^{p}\;\neq \;\Omega^{p}.$$

In addition, if the total force density supply and the total momentum supply are zero according to Equation (62)${}_{2}$,

(68) $$T^{kl}{}_{;k}\;=\;0\;⟶\;\Omega_{\mathrm{iso}}\;=\;0\;\wedge \;\Omega_{\mathrm{iso}}^{m}\;=\;0,$$

(∧: the conjunction of the formal logic) we obtain according to Equation (64)${}_{1,2}$

(69) $$\begin{array}{ccc} \sum_{A}\Omega_{\mathrm{iso}}^{Am}u_{m} & = & -\sum_{A}f^{A}\Omega_{\mathrm{iso}}^{A}\;\neq \;0, \end{array}$$

(70) $$\begin{array}{ccc} \sum_{A}\Omega_{\mathrm{iso}}^{Am}h_{m}^{p} & = & -\sum_{A}g^{Ap}\Omega_{\mathrm{iso}}^{A}\;\neq \;0. \end{array}$$

As expected, the supplies of energy and momentum remain entangled in a system of vanishing total force and momentum densities. The entanglement vanishes for such isolated systems for which the force and momentum supplies for all ${}^{A}$-components are zero.

## 5. The Spin Tensor

### 5.1. (3+1)-Split

The (3+1)-split of the spin tensor of an ${}^{A}$-component is defined by inserting Equation (19)${}_{1}$ into

(71) $$S^{Akab}\;=\;S^{Ampq}\delta_{m}^{k}\delta_{p}^{a}\delta_{q}^{b}.$$

Introducing the following covariant abbreviations

(72) $$\begin{array}{c} s^{Amj}\;:=\;S^{Akab}u_{k}^{A}h_{a}^{Am}h_{b}^{Aj},\;s^{Amji}\;:=\;S^{Akab}h_{k}^{Am}h_{a}^{Aj}h_{b}^{Ai} \end{array}$$

(73) $$\begin{array}{c} \Xi^{Am}\;:=\;S^{Akab}u_{k}^{A}u_{a}^{A}h_{b}^{Am},\;\Xi^{Amj}\;:=\;S^{Akab}h_{k}^{Am}u_{a}^{A}h_{b}^{Aj}, \end{array}$$

Equation (71) results in

(74) $$\begin{array}{c} S^{Akab}\;=\;-S^{Akba}\;=\;\;\\ =\;u^{Aa}\left(\frac{1}{c^{4}}u^{Ak}\Xi^{Ab}+\frac{1}{c^{2}}\Xi^{Akb}\right)-u^{Ab}\left(\frac{1}{c^{4}}u^{Ak}\Xi^{Aa}+\frac{1}{c^{2}}\Xi^{Aka}\right)+s^{Akab}+\frac{1}{c^{2}}u^{Ak}s^{Aab}.\; \end{array}$$

By Equations (72) and (73), the following quantities are introduced: the spin density $s^{ab}$, the spin density vector $\Xi^{b}$, the couple stress $s^{kab}$ and the spin stress $\Xi^{kb}$.

Analogously to Equations (40) and (41), a more compact form of the spin tensor is

(75) $$\begin{array}{cc} & S^{Akab}\;=\;2u^{A[a}L^{Akb]}+M^{Akab}, \end{array}$$

(76) $$\begin{array}{cc} L^{Akb}\;:=\;\frac{1}{c^{4}}u^{Ak}\Xi^{Ab}+\frac{1}{c^{2}}\Xi^{Akb},\; & M^{Akab}\;:=\;s^{Akab}+\frac{1}{c^{2}}u^{Ak}s^{Aab}. \end{array}$$

Taking Equations (72) and (73) into account, we obtain

(77) $$S^{Akab}u_{a}^{A}\;=\;c^{2}L^{Akb},\;S^{Akab}h_{a}^{Am}h_{b}^{An}\;=\;M^{Akmn},$$

expressions which are needed for formulating the entropy identity below.

### 5.2. Additivity

Analogously to Setting II, we introduce the spin tensor of the mixture as the sum of the spin tensors of the ${}^{A}$-components.

(78) $$\begin{array}{ccc} \mathrm{Setting}\mathrm{III}:\\ S^{kab} & := & 2u^{[a}L^{kb]}+M^{kab}\overset{•}{=}\;\sum_{A}S^{Akab}\;=\;\sum_{A}\left(2u^{A[a}L^{Akb]}+M^{Akab}\right)\;=\;\\ & = & \sum_{A}\left\{2u^{A[a}\left(\frac{1}{c^{4}}u^{Ak}\Xi^{Ab]}+\frac{1}{c^{2}}\Xi^{Akb]}\right)+s^{Akab}+\frac{1}{c^{2}}u^{Ak}s^{Aab}.\right\}. \end{array}$$

According to the mixture axiom, the spin tensor of the mixture is defined by Equation (78)${}_{1}$ as spin of a 1-component system resulting from Equation (75) by setting A≡1→blank.

### 5.3. (3+1)-Components of the Mixture

From Equation (78)${}_{1}$, we obtain by taking the mixture axiom and Equation (77) into account

(79) $$\begin{array}{ccc} S^{kab}u_{a} & = & c^{2}L^{ab}\;=\;\sum_{A}\left(2u^{A[a}L^{Akb]}+M^{Akab}\right)u_{a}, \end{array}$$

(80) $$\begin{array}{ccc} S^{kab}h_{a}^{m}h_{b}^{n} & = & M^{kmn}\;=\;\sum_{A}\left(2u^{A[a}L^{Akb]}+M^{Akab}\right)h_{a}^{m}h_{b}^{n}. \end{array}$$

The (3+1)-components of the spin tensor of the mixture result from Equations (72) and (73) using the mixture axiom

(81) $$\begin{array}{c} s^{mj}\;=\;S^{kab}u_{k}h_{a}^{m}h_{b}^{j},\;s^{mji}\;=\;S^{kab}h_{k}^{m}h_{a}^{j}h_{b}^{i} \end{array}$$

(82) $$\begin{array}{c} \Xi^{m}\;=\;S^{kab}u_{k}u_{a}h_{b}^{m},\;\Xi^{mj}\;=\;S^{kab}h_{k}^{m}u_{a}h_{b}^{j}, \end{array}$$

and by inserting Equation (78)${}_{4}$ or Equation (79) and Equation (80).

### 5.4. Spin Balance Equation

If there exists an external angular momentum density

(83) $$m^{ab}\;=\;-m^{ba},$$

a spin balance equation of each ${}^{A}$-component and of the mixture has to be taken into account

(84) $$S^{Akab}{}_{;k}\;=\;\frac{1}{c^{2}}m^{Aab},\;S^{kab}{}_{;k}\;=\;\frac{1}{c^{2}}m^{ab}.$$

According to Setting III,

(85) $$\sum_{A}m^{Aab}\;=\;m^{ab}$$

the additivity of the partial angular momenta is valid.

## 6. Thermodynamics of Interacting Components

### 6.1. The Entropy Identity

Starting with the (3+1)-split of the entropy 4-vector and the entropy balance equation

(86) $$\begin{array}{ccc} S^{Ak}\;=\;s^{A}u^{Ak}+s^{Ak}\; & ⟶ & S^{Ak}{}_{;k}\;=\;\sigma^{A}+\varphi^{A}, \end{array}$$

(87) $$\begin{array}{ccc} s^{A}\;:=\;\frac{1}{c^{2}}S^{Ak}u_{k}^{A}, & \; & s^{Ak}\;:=\;S^{Am}h_{m}^{Ak} \end{array}$$

we have to define the following four quantities in accordance with the balance equations of mass in Equation (1)${}_{2}$, of energy in Equation (26), of momentum in Equation (27) and of spin in Equation (84)${}_{1}$: the entropy density $s^{A}$, the entropy flux density $s^{Ak}$, the entropy production $\sigma^{A}$ and the entropy supply $\varphi^{A}$. Because there is no unequivocal entropy [15] and, consequently, also no unique entropy density, entropy-flux, entropy-production and entropy-supply, we need a tool which helps to restrict the arbitrariness for defining entropies. Such a tool is the *entropy identity* [10,11], which is generated by adding suitable zeros to the entropy in Equation (86)${}_{1}$, which are related to the balances that are taken into account. These zeros are generated by choosing the following expressions: $N^{Ak},\;u_{l}^{A}T^{Akl},\;h_{l}^{Am}T^{Akl},\;u_{a}^{A}S^{Akab},h_{a}^{Am}h_{b}^{An}S^{Akab}$. Consequently according to Equations (1), (41) and (77), the entropy identity is chosen as

(88) $$\begin{array}{ccc} S^{Ak} & \equiv & s^{A}u^{Ak}+s^{Ak}+\kappa^{A}\left(N^{Ak}-\varrho^{A}u^{Ak}\right)+\\ & & +\lambda^{A}\left(u_{l}^{A}T^{Akl}-e^{A}u^{Ak}-q^{Ak}\right)+\\ & & +\lambda_{m}^{A}\left(h_{l}^{Am}T^{Akl}-u^{Ak}p^{Am}-t^{Akm}\right)+\\ & & +\Lambda_{m}^{A}\left(u_{a}^{A}h_{b}^{Am}S^{Akab}-\frac{1}{c^{2}}u^{Ak}\Xi^{Am}-\Xi^{Akm}\right)+\\ & & +\Lambda_{mn}^{A}\left(h_{a}^{Am}h_{b}^{An}S^{Akab}-s^{Akmn}-\frac{1}{c^{2}}u^{Ak}s^{Amn}\right). \end{array}$$

The fields of Lagrange multipliers $\kappa^{A},\;\lambda^{A},\;\lambda_{m}^{A}$, $\Lambda_{m}^{A}$ and $\Lambda_{mn}^{A}$ are quantities whose physical meaning becomes clear in the course of the exploitation of the entropy identity. Here, $\kappa^{A}$ and $\lambda^{A}$ are scalars, undefined for the present, and for the likewise arbitrary quantities $\lambda_{m}^{A}$, $\Lambda_{m}^{A}$ and $\Lambda_{ab}^{A}$, tensors of first and second order. An identification of these Lagrange multipliers is given below after the definitions of entropy flux density, entropy production density and supply in Section 6.3.

The entropy identity in Equation (89) depends on the balances which are taken into consideration as constraints: the balances of mass, energy, momentum and spin. The electro-magnetic field and quantum fields are included, if the energy–momentum tensor and the spin tensor of these fields are inserted into Equation (89).

Considering the third, fourth and fifth rows of Equation (89), we obtain that the velocity parts of $\lambda_{m}^{A}$, $\Lambda_{m}^{A}$ and $\Lambda_{mn}^{A}$ can be set to zero according to Equations (72) and (73). The symmetric part of $\Lambda_{mn}^{A}$ does not contribute to the fifth row of Equation (89) and therefore it is set to zero, too

(89) $$\lambda_{m}^{A}h_{l}^{Am}\;=\;\lambda_{l}^{A},\;\Lambda_{m}^{A}h_{b}^{Am}\;=\;\Lambda_{b}^{A},\;\Lambda_{mn}^{A}h_{a}^{Am}h_{b}^{An}\;=\;\Lambda_{ab}^{A}\;=\;-\Lambda_{ba}^{A}.$$

The entropy identity in Equation (89) becomes by rearranging

(90) $$\begin{array}{ccc} S^{Ak} & \equiv & u^{Ak}\left(s^{A}-\kappa^{A}\varrho^{A}-\lambda^{A}e^{A}-\lambda_{m}^{A}p^{Am}-\Lambda_{m}^{A}\frac{1}{c^{2}}\Xi^{Am}-\Lambda_{mn}^{A}\frac{1}{c^{2}}s^{Amn}\right)+\\ & & +s^{Ak}+\kappa^{A}N^{Ak}+\left(\lambda^{A}u_{l}^{A}+\lambda_{l}^{A}\right)T^{Akl}-\lambda^{A}q^{Ak}-\lambda_{l}^{A}t^{Akl}+\\ & & +\left(\Lambda_{b}^{A}u_{a}^{A}+\Lambda_{ab}^{A}\right)S^{Akab}-\Lambda_{m}^{A}\Xi^{Akm}-\Lambda_{mn}^{A}s^{Akmn}. \end{array}$$

This identity transforms into an other one by differentiation and by taking the balance equations of mass in Equation (1)${}_{2}$, of energy–momentum in Equation (25), of spin in Equation (84)${}_{1}$ and of entropy in Equation (86)${}_{2}$ into account.

(91) $$\begin{array}{ccc} S^{Ak}{}_{;k} & \equiv & {\left[u^{Ak}\left(s^{A}-\kappa^{A}\varrho^{A}-\lambda^{A}e^{A}-\lambda_{m}^{A}p^{Am}-\Lambda_{m}^{A}\frac{1}{c^{2}}\Xi^{Am}-\Lambda_{mn}^{A}\frac{1}{c^{2}}s^{Amn}\right)\right]}_{;k}+\\ & & +s^{Ak}{}_{;k}+\kappa^{A}{}_{;k}N^{Ak}+\kappa^{A}\left({}^{\left(ex\right)}\Gamma^{A}{+}^{\left(in\right)}\;\Gamma^{A}\right)+\\ & & +{\left(\lambda^{A}u_{l}^{A}+\lambda_{l}^{A}\right)}_{;k}T^{Akl}+\left(\lambda^{A}u_{l}^{A}+\lambda_{l}^{A}\right)k^{Al}-\\ & & -\left(\lambda^{A}q^{Ak}\right){}_{;k}-\left(\lambda_{l}^{A}t^{Akl}\right){}_{;k}+\\ & & +{\left(\Lambda_{b}^{A}u_{a}^{A}+\Lambda_{ab}^{A}\right)}_{;k}S^{Akab}+\left(\Lambda_{b}^{A}u_{a}^{A}+\Lambda_{ab}^{A}\right)\frac{1}{c^{2}}m^{Aab}-\\ & & -{\left(\Lambda_{m}^{A}\Xi^{Akm}\right)}_{;k}-{\left(\Lambda_{mn}^{A}s^{Akmn}\right)}_{;k}\;=\;\sigma^{A}+\varphi^{A}. \end{array}$$

Here, $\sigma^{A}$ is the entropy production and $\varphi^{A}$ the entropy supply of the ${}^{A}$-component. The identity in Equation (91) changes into the entropy production, if $s^{A}$, $s^{Ak}$ and $\varphi^{A}$ are specified below.

Rearranging the entropy identity results in

(92) $$\begin{array}{ccc} S^{Ak}{}_{;k} & \equiv & u^{Ak}{}_{;k}\left(s^{A}-\kappa^{A}\varrho^{A}-\lambda^{A}e^{A}-\lambda_{m}^{A}p^{Am}-\Lambda_{m}^{A}\frac{1}{c^{2}}\Xi^{Am}-\Lambda_{mn}^{A}\frac{1}{c^{2}}s^{Amn}\right)+\\ & & +u^{Ak}{\left(s^{A}-\kappa^{A}\varrho^{A}-\lambda^{A}e^{A}-\lambda_{m}^{A}p^{Am}-\Lambda_{m}^{A}\frac{1}{c^{2}}\Xi^{Am}-\Lambda_{mn}^{A}\frac{1}{c^{2}}s^{Amn}\right)}_{;k}+\\ & & +{\left(s^{Ak}-\lambda^{A}q^{Ak}-\lambda_{l}^{A}t^{Akl}-\Lambda_{m}^{A}\Xi^{Akm}-\Lambda_{mn}^{A}s^{Akmn}\right)}_{;k}+\\ & & +\kappa^{A}{}^{\left(ex\right)}\Gamma^{A}+\left(\lambda^{A}u_{l}^{A}+\lambda_{l}^{A}\right)k^{Al}+\left(\Lambda_{b}^{A}u_{a}^{A}+\Lambda_{ab}^{A}\right)\frac{1}{c^{2}}m^{Aab}+\\ & & +\kappa^{A}{}_{;k}N^{Ak}+\kappa^{A}{}^{\left(in\right)}\;\Gamma^{A}+\left(\lambda^{A}u_{l}^{A}+\lambda_{l}^{A}\right){}_{;k}T^{Akl}+{\left(\Lambda_{b}^{A}u_{a}^{A}+\Lambda_{ab}^{A}\right)}_{;k}S^{Akab}\;=\;\;\\ & & =\;\sigma^{A}+\varphi^{A}. \end{array}$$

Now, we look for terms of the fifth row of Equation (92) which fit into the first three rows of Equation (92). The shape of these terms is $\left[u^{Ak}{}_{;k}\mathrm{scalar}/u^{Ak}\mathrm{scalar}{}_{;k}\right]$ according to the first two rows of Equation (92) and $\left[\Psi^{Ak}{}_{;k}\;\left(\Psi^{Ak}u_{k}^{A}=0\right)\right]$ according to the third row. None of the seven terms of the fourth and fifth rows of Equation (92) have this shape, but inserting the energy–momentum tensor and the spin tensor into the fifth row of Equation (92) may generate such terms.

The third term of the fifth row of Equation (92) becomes

$$\begin{array}{ccc} {\left(\lambda^{A}u_{l}^{A}\right)}_{;k}T^{Akl}\; & = & \;\left(\lambda^{A}{}_{;k}u_{l}^{A}+\lambda^{A}u^{A}{}_{l;k}\right)\left(\frac{1}{c^{2}}e^{A}u^{Ak}u^{Al}+u^{Ak}p^{Al}+\frac{1}{c^{2}}q^{Ak}u^{Al}+t^{Akl}\right)= \end{array}$$

(93) $$\begin{array}{ccc} & = & \;\lambda {}^{A}{}_{;k}u^{Ak}e^{A}+\lambda^{A}u^{A}{}_{l;k}u^{Ak}p^{Al}+\lambda^{A}{}_{;k}q^{Ak}-\underline{p^{A}\lambda^{A}u^{Ak}{}_{;k}}+\lambda^{A}u^{A}{}_{l;k}\pi^{Akl},\; \end{array}$$

(94) $$\begin{array}{ccc} \lambda^{A}{}_{l;k}T^{Akl}\; & = & \;\lambda^{A}{}_{l;k}\left(\frac{1}{c^{2}}e^{A}u^{Ak}u^{Al}+u^{Ak}p^{Al}+\frac{1}{c^{2}}q^{Ak}u^{Al}+t^{Akl}\right) \end{array}$$

Summing up Equations (93) and (94) results in (the signs $\underline{⊡}$, $\overset{︷}{⊡}$, $\underset{︸}{⊡}$ and $\tilde{⊡}$ mark terms which are related to each other in the sequel)

(95) $$\begin{array}{c} {\left(\lambda^{A}u_{l}^{A}+\lambda_{l}^{A}\right)}_{;k}T^{Akl}\;=\;\lambda {}^{A}{}_{;k}\left(q^{Ak}+e^{A}u^{Ak}\right)+\;\lambda^{A}u^{A}{}_{l;k}\left(\pi^{Akl}+u^{Ak}p^{Al}\right)-\\ -\underline{p^{A}\lambda^{A}u^{Ak}{}_{;k}}+\;\lambda^{A}{}_{l;k}\left(\frac{1}{c^{2}}e^{A}u^{Ak}u^{Al}+u^{Ak}p^{Al}+\frac{1}{c^{2}}q^{Ak}u^{Al}+t^{Akl}\right).\; \end{array}$$

Evidently, the term $-\underline{p^{A}\lambda^{A}u^{Ak}{}_{;k}}$ belongs to the first row of Equation (92). After having inserted the underlined term of Equation (95), the first two rows of Equation (92) become (${}^{•}$ is the “component time derivative” $\overset{•}{⊞}{}^{A}:={⊞}^{A}{}_{;k}u^{Ak}$)

(96) $$\begin{array}{c} u^{Ak}{}_{;k}\left(s^{A}-\kappa^{A}\varrho^{A}-\lambda^{A}e^{A}-\underline{p^{A}\lambda^{A}}-\lambda_{m}^{A}p^{Am}-\Lambda_{m}^{A}\frac{1}{c^{2}}\Xi^{Am}-\Lambda_{mn}^{A}\frac{1}{c^{2}}s^{Amn}\right)+\\ +\left(s^{A}-\kappa^{A}\varrho^{A}-\lambda^{A}e^{A}-\underset{︸}{p^{A}\lambda^{A}}-\lambda_{m}^{A}p^{Am}-\Lambda_{m}^{A}\frac{1}{c^{2}}\Xi^{Am}-\Lambda_{mn}^{A}\frac{1}{c^{2}}s^{Amn}\right){}^{•}+\underset{︸}{\left(p^{A}\lambda^{A}\right){}^{•}}=\;\\ =\;{\left[u^{Ak}\left(s^{A}-\kappa^{A}\varrho^{A}-\lambda^{A}e^{A}-p^{A}\lambda^{A}-\lambda_{m}^{A}p^{Am}-\Lambda_{m}^{A}\frac{1}{c^{2}}\Xi^{Am}-\Lambda_{mn}^{A}\frac{1}{c^{2}}s^{Amn}\right)\right]}_{;k}+\\ +\tilde{\left(p^{A}\lambda^{A}\right){}^{•}}. \end{array}$$

Thus, a rearranging of the entropy identity in Equation (92) results by taking Equation (96) into account

(97) $$\begin{array}{cc} & S^{Ak}{}_{;k}\;\equiv \\ & {\left[u^{Ak}\left(s^{A}-\kappa^{A}\varrho^{A}-\lambda^{A}e^{A}-p^{A}\lambda^{A}-\lambda_{m}^{A}p^{Am}-\Lambda_{m}^{A}\frac{1}{c^{2}}\Xi^{Am}-\Lambda_{mn}^{A}\frac{1}{c^{2}}s^{Amn}\right)\right]}_{;k}+\;\\ & +\tilde{\left(p^{A}\lambda^{A}\right){}^{•}}+\left(s^{Ak}-\lambda^{A}q^{Ak}-\lambda_{l}^{A}t^{Akl}-\Lambda_{m}^{A}\Xi^{Akm}-\Lambda_{mn}^{A}s^{Akmn}\right){}_{;k}+\\ & +\kappa^{A}{}^{\left(ex\right)}\Gamma^{A}+\left(\lambda^{A}u_{l}^{A}+\lambda_{l}^{A}\right)k^{Al}+\left(\Lambda_{b}^{A}u_{a}^{A}+\Lambda_{ab}^{A}\right)\frac{1}{c^{2}}m^{Aab}+\\ & +\kappa^{A}{}_{;k}N^{Ak}+\kappa^{A}{}^{\left(in\right)}\;\Gamma^{A}+{\left(\Lambda_{b}^{A}u_{a}^{A}+\Lambda_{ab}^{A}\right)}_{;k}S^{Akab}+\\ & +\lambda {}^{A}{}_{;k}\left(q^{Ak}+e^{A}u^{Ak}\right)+\;\lambda^{A}u^{A}{}_{l;k}\left(\pi^{Akl}+u^{Ak}p^{Al}\right)+\\ & +\;\overset{•}{\lambda }{}_{l}^{A}\left(\frac{1}{c^{2}}e^{A}u^{Al}+p^{Al}\right)+\lambda^{A}{}_{l;k}\left(\frac{1}{c^{2}}q^{Ak}u^{Al}+t^{Akl}\right)\;=\;\sigma^{A}+\varphi^{A}.\; \end{array}$$

The third term of the fourth row of Equation (97) results in

(98) $$\begin{array}{c} {\left(\Lambda_{b}^{A}u_{a}^{A}+\Lambda_{ab}^{A}\right)}_{;k}S^{Akab}\;=\;\\ =\left(\Lambda_{b;k}^{A}u_{a}^{A}+\Lambda_{b}^{A}u_{a;k}^{A}+\Lambda_{ab;k}^{A}\right)\left(u^{Aa}L^{Akb}-u^{Ab}L^{Aka}+M^{Akab}\right). \end{array}$$

If Equations (89) and (76) are taken into account, these nine terms are:

(99) $$\begin{array}{ccc} \Lambda_{b;k}^{A}c^{2}L^{Akb} & = & \Lambda_{b;k}^{A}\left(\frac{1}{c^{2}}u^{Ak}\Xi^{Ab}+\Xi^{Akb}\right), \end{array}$$

(100) $$\begin{array}{ccc} \Lambda_{b}^{A}\underset{︸}{u_{a;k}^{A}}\underset{︸}{u^{Aa}}L^{Akb} & = & 0, \end{array}$$

(101) $$\begin{array}{ccc} \Lambda_{ab;k}^{A}u^{Aa}L^{Akb} & = & \Lambda_{ab;k}^{A}u^{Aa}\left(\frac{1}{c^{4}}u^{Ak}\Xi^{Ab}+\frac{1}{c^{2}}\Xi^{Akb}\right), \end{array}$$

(102) $$\begin{array}{ccc} -\Lambda_{b;k}^{A}u_{a}^{A}u^{Ab}L^{Aka} & = & -\Lambda_{b;k}^{A}\underset{︸}{u_{a}^{A}}u^{Ab}\left(\frac{1}{c^{4}}u^{Ak}\underset{︸}{\Xi^{Aa}}+\frac{1}{c^{2}}\underset{︸}{\Xi^{Aka}}\right)\;=\;0, \end{array}$$

(103) $$\begin{array}{ccc} -\underset{︸}{\Lambda_{b}^{A}}u_{a;k}^{A}\underset{︸}{u^{Ab}}L^{Aka} & = & 0, \end{array}$$

(104) $$\begin{array}{ccc} -\Lambda_{ab;k}^{A}u^{Ab}L^{Aka} & = & -\Lambda_{ab;k}^{A}u^{Ab}\left(\frac{1}{c^{4}}u^{Ak}\Xi^{Aa}+\frac{1}{c^{2}}\Xi^{Aka}\right), \end{array}$$

(105) $$\begin{array}{ccc} \Lambda_{b;k}^{A}\underset{︸}{u_{a}^{A}}\underset{︸}{M^{Akab}} & = & 0, \end{array}$$

(106) $$\begin{array}{ccc} \Lambda_{b}^{A}u_{a;k}^{A}M^{Akab} & = & \Lambda_{b}^{A}u_{a;k}^{A}\left(s^{Akab}+\frac{1}{c^{2}}u^{Ak}s^{Aab}\right), \end{array}$$

(107) $$\begin{array}{ccc} \Lambda_{ab;k}^{A}M^{Akab} & = & \Lambda_{ab;k}^{A}\left(s^{Akab}+\frac{1}{c^{2}}u^{Ak}s^{Aab}\right). \end{array}$$

Rearranging of Equations (99)–(107) results in:

Equations (99) and (106):

(108) $$\begin{array}{c} \overset{•}{\Lambda }{}_{b}^{A}\frac{1}{c^{2}}\Xi^{Ab}+\Lambda_{b;k}^{A}\Xi^{Akb}-\Lambda_{b}^{A}u_{a}^{A}\left(s^{Akab}{}_{;k}+\frac{1}{c^{2}}\overset{•}{s}\;{}^{Aab}\right), \end{array}$$

Equations (101), (104) and (107):

(109) $$\begin{array}{c} \overset{•}{\Lambda }\;{}_{ab}^{A}\left(\frac{1}{c^{4}}u^{A[a}\Xi^{Ab]}+\frac{1}{c^{2}}s^{Aab}\right)+\Lambda {}_{ab;k}^{A}\left(\frac{1}{c^{2}}u^{A[a}\Xi^{Akb]}+s^{Akab}\right). \end{array}$$

A comparison of Equations (108) and (109) with the first two rows of Equation (97) demonstrates that a term which fits into these rows does not appear in Equations (108) and (109). Thus, by taking Equation (96) into account, a rearranging of the entropy identity in Equation (92) results in

(110) $$\begin{array}{ccc} S^{Ak}{}_{;k} & \;\equiv & \\ & & {\left[u^{Ak}\left(s^{A}-\kappa^{A}\varrho^{A}-\lambda^{A}e^{A}-p^{A}\lambda^{A}-\lambda_{m}^{A}p^{Am}-\Lambda_{m}^{A}\frac{1}{c^{2}}\Xi^{Am}-\Lambda_{mn}^{A}\frac{1}{c^{2}}s^{Amn}\right)\right]}_{;k}+\;\\ & & +\tilde{\left(p^{A}\lambda^{A}\right){}^{•}}+{\left(s^{Ak}-\lambda^{A}q^{Ak}-\lambda_{l}^{A}t^{Akl}-\Lambda_{m}^{A}\Xi^{Akm}-\Lambda_{mn}^{A}s^{Akmn}\right)}_{;k}+\\ & & +\kappa^{A}{}^{\left(ex\right)}\Gamma^{A}+\left(\lambda^{A}u_{l}^{A}+\lambda_{l}^{A}\right)k^{Al}+\left(\Lambda_{b}^{A}u_{a}^{A}+\Lambda_{ab}^{A}\right)\frac{1}{c^{2}}m^{Aab}+\\ & & +\kappa^{A}{}_{;k}N^{Ak}+\kappa^{A}{}^{\left(in\right)}\;\Gamma^{A}+\lambda {}^{A}{}_{;k}\left(q^{Ak}+e^{A}u^{Ak}\right)+\;\lambda^{A}u^{A}{}_{l;k}\left(\pi^{Akl}+u^{Ak}p^{Al}\right)+\\ & & +\;\overset{•}{\lambda }{}_{l}^{A}\left(\frac{1}{c^{2}}e^{A}u^{Al}+p^{Al}\right)+\lambda^{A}{}_{l;k}\left(\frac{1}{c^{2}}q^{Ak}u^{Al}+t^{Akl}\right)+\\ & & +\overset{•}{\Lambda }{}_{b}^{A}\frac{1}{c^{2}}\Xi^{Ab}+\Lambda_{b;k}^{A}\Xi^{Akb}-\Lambda_{b}^{A}u_{a}^{A}\left(s^{Akab}{}_{;k}+\frac{1}{c^{2}}\overset{•}{s}\;{}^{Aab}\right)+\\ & & +\overset{•}{\Lambda }\;{}_{ab}^{A}\left(\frac{1}{c^{4}}u^{A[a}\Xi^{Ab]}+\frac{1}{c^{2}}s^{Aab}\right)+\Lambda {}_{ab;k}^{A}\left(\frac{1}{c^{2}}u^{A[a}\Xi^{Akb]}+s^{Akab}\right)\;=\\ & & =\;\sigma^{A}+\varphi^{A}. \end{array}$$

This entropy identity is incomplete: the multi-temperature relaxation is missing which is generated by the different partial temperatures of the components of the mixture. Because of lucidity, the treatment of multi-temperature relaxation is postponed and is considered in Section 6.4. In the next section, we now specify $s^{A}$, $s^{Ak}$, $\varphi^{A}$ and $\sigma^{A}$.

### 6.2. Exploitation of the Entropy Identity

#### 6.2.1. Entropy Density, Gibbs and Gibbs–Duhem Equations

We now define the *entropy rest density* $s^{A}$ according to the first row of Equation (110)

(111) $$\begin{array}{c} \mathrm{Setting}\mathrm{IV}:\;\\ s^{A}\;\overset{•}{=}\;\kappa^{A}\varrho^{A}+\lambda^{A}e^{A}+p^{A}\lambda^{A}+\lambda_{l}^{A}p^{Al}+\Lambda_{m}^{A}\frac{1}{c^{2}}\Xi^{Am}+\Lambda_{mn}^{A}\frac{1}{c^{2}}s^{Amn}, \end{array}$$

resulting in the specific rest entropy

(112) $$\frac{s^{A}}{\varrho }\;=\;\kappa^{A}\frac{\varrho^{A}}{\varrho }+\lambda^{A}\frac{e^{A}}{\varrho }+p^{A}\lambda^{A}\frac{1}{\varrho }+\lambda_{l}^{A}\frac{p^{Al}}{\varrho }+\Lambda_{m}^{A}\frac{1}{c^{2}}\frac{\Xi^{Am}}{\varrho }+\Lambda_{mn}^{A}\frac{1}{c^{2}}\frac{s^{Amn}}{\varrho }.$$

A non-equilibrium state space—which is spanned by the independent variables—contains, besides $\varrho^{A}$, ϱ, and $e^{A}$, the spin variables $\Xi^{Am}$ and $s^{Amn}$ as well as $p^{Al}$, which extends the state space in the sense of Extended Thermodynamics (if the energy–momentum tensor is presupposed to be symmetric—consequently $p^{Al}=\left(1/c^{2}\right)q^{Al}$ is valid according to Equation (33)—the momentum density is replaced by energy flux density which in non-relativistic Extended Thermodynamics is set as a non-equilibrium variable, even if the stress tensor is non-symmetric) [19,20]. Consequently, we choose the *state space* [21]

(113) $$z^{A}\;=\;\left(c^{A},\frac{1}{\varrho },\frac{e^{A}}{\varrho },\frac{p^{Al}}{\varrho },\frac{\Xi^{Am}}{\varrho },\frac{s^{Amn}}{\varrho }\right),\;c^{A}\;:=\;\frac{\varrho^{A}}{\varrho },$$

introducing the concentration $c^{A}$.

The corresponding *Gibbs equation* according to Equations (112) and (113) is

(114) $$\begin{array}{c} {\left(\frac{s^{A}}{\varrho }\right)}^{{}^{•}}\;=\;\kappa^{A}\overset{•}{c}{}^{A}+\lambda^{A}{\left(\frac{e^{A}}{\varrho }\right)}^{{}^{•}}+p^{A}\lambda^{A}{\left(\frac{1}{\varrho }\right)}^{{}^{•}}+\lambda_{l}^{A}{\left(\frac{p^{Al}}{\varrho }\right)}^{{}^{•}}+\\ +\Lambda_{m}^{A}\frac{1}{c^{2}}{\left(\frac{\Xi^{Am}}{\varrho }\right)}^{{}^{•}}+\Lambda_{mn}^{A}\frac{1}{c^{2}}{\left(\frac{s^{Amn}}{\varrho }\right)}^{{}^{•}} \end{array}$$

Differentiation of Equation (112) results in the *Gibbs–Duhem equation* by taking (114) into account

(115) $$0\;=\;\overset{•}{\kappa }\;{}^{A}c^{A}+\overset{•}{\lambda }\;{}^{A}\frac{e^{A}}{\varrho }+{\left(p^{A}\lambda^{A}\right)}^{{}^{•}}\frac{1}{\varrho }+\overset{•}{\lambda }{}_{l}^{A}\frac{p^{Al}}{\varrho }+\overset{•}{\Lambda }\;{}_{m}^{A}\frac{1}{c^{2}}\frac{\Xi^{Am}}{\varrho }+\overset{•}{\Lambda }\;{}_{mn}^{A}\frac{1}{c^{2}}\frac{s^{Amn}}{\varrho },$$

resulting in

(116) $$\tilde{{\left(p^{A}\lambda^{A}\right)}^{•}}\;=\;-\overset{•}{\kappa }\;{}^{A}\varrho^{A}-\overset{•}{\lambda }\;{}^{A}e^{A}-\overset{•}{\lambda }{}_{l}^{A}p^{Al}-\overset{•}{\Lambda }\;{}_{m}^{A}\frac{1}{c^{2}}\Xi^{Am}-\overset{•}{\Lambda }\;{}_{mn}^{A}\frac{1}{c^{2}}s^{Amn}.$$

Taking Equations (116) and (111) into account, the entropy identity in Equation (110) becomes

(117) $$\begin{array}{cc} S^{Ak}{}_{;k}\equiv & -\underset{︸}{\overset{•}{\kappa }\;{}^{A}\varrho^{A}}-\underline{\overset{•}{\lambda }\;{}^{A}e^{A}}-\hat{\overset{•}{\lambda }{}_{l}^{A}p^{Al}}-\tilde{\overset{•}{\Lambda }\;{}_{m}^{A}\frac{1}{c^{2}}\Xi^{Am}}-\overset{︷}{\overset{•}{\Lambda }\;{}_{mn}^{A}\frac{1}{c^{2}}s^{Amn}}+\\ & +\;{\left(\;s^{Ak}-\lambda^{A}q^{Ak}-\lambda_{l}^{A}t^{Akl}-\Lambda_{m}^{A}\Xi^{Akm}-\Lambda_{mn}^{A}s^{Akmn}\right)}_{;k}+\\ & +\kappa^{A}{}^{\left(ex\right)}\Gamma^{A}+\left(\lambda^{A}u_{l}^{A}+\lambda_{l}^{A}\right)k^{Al}+\left(\Lambda_{b}^{A}u_{a}^{A}+\Lambda_{ab}^{A}\right)\frac{1}{c^{2}}m^{Aab}+\\ & +\underset{︸}{\kappa^{A}{}_{;k}N^{Ak}}+\kappa^{A}{}^{\left(in\right)}\;\Gamma^{A}+\underline{\lambda {}^{A}{}_{;k}}\left(q^{Ak}+\underline{e^{A}u^{Ak}}\right)+\;\lambda^{A}u^{A}{}_{l;k}\left(\pi^{Akl}+u^{Ak}p^{Al}\right)+\\ & +\;\hat{\overset{•}{\lambda }{}_{l}^{A}}\left(\frac{1}{c^{2}}e^{A}u^{Al}+\hat{p^{Al}}\right)+\lambda^{A}{}_{l;k}\left(\frac{1}{c^{2}}q^{Ak}u^{Al}+t^{Akl}\right)+\\ & +\tilde{\overset{•}{\Lambda }{}_{b}^{A}\frac{1}{c^{2}}\Xi^{Ab}}+\Lambda_{b;k}^{A}\Xi^{Akb}-\Lambda_{b}^{A}u_{a}^{A}\left(s^{Akab}{}_{;k}+\frac{1}{c^{2}}\overset{•}{s}\;{}^{Aab}\right)+\\ & +\overset{︷}{\overset{•}{\Lambda }\;{}_{ab}^{A}}\left(\frac{1}{c^{4}}u^{A[a}\Xi^{Ab]}+\overset{︷}{\frac{1}{c^{2}}s^{Aab}}\right)+\Lambda {}_{ab;k}^{A}\left(\frac{1}{c^{2}}u^{A[a}\Xi^{Akb]}+s^{Akab}\right)\;=\\ & =\;\sigma^{A}+\varphi^{A}. \end{array}$$

The marked terms cancel each other.

Taking Equations (15)${}_{2}$ and (22)${}_{2}$ into account, we consider

(118) $$\begin{array}{ccc} 0 & = & \underset{︸}{\kappa^{A}{}_{;k}N^{Ak}}-\underset{︸}{\overset{•}{\kappa }\;{}^{A}\varrho^{A}}\;=\;\kappa^{A}{}_{;k}\left(N^{Ak}-N^{Ak}\right)\;=\;\\ & = & \kappa^{A}{}_{;k}\left(J^{Ak}+\varrho^{A}f^{A}u^{k}-J^{Ak}-\varrho^{A}f^{A}u^{k}\right)\;=\;\kappa^{A}{}_{;k}\left(J^{Ak}-J^{Ak}\right)\;=\;\\ & = & \kappa^{A}{}_{;k}J^{Ak}-\kappa^{A}{}_{;k}\left(J^{Am}h_{m}^{Ak}+w^{A}N^{Ak}\right)\;=\;\\ & = & \kappa^{A}{}_{;k}\left(\underset{J^{Am}h_{m}^{Ak}}{\underset{︸}{J^{Ak}-w^{A}N^{Ak}}}\right)-{\left(\kappa^{A}J^{Am}h_{m}^{Ak}\right)}_{;k}+\kappa^{A}{\left(J^{Am}h_{m}^{Ak}\right)}_{;k} \end{array}$$

This zero contains the diffusion flux which does not appear in the entropy identity in Equation (89) up to here. That means the diffusion is missing in Equation (117), and we do not ignore the underbraced terms in Equation (118)${}_{1}$, but we insert Equation (118)${}_{3}$ into Equation (117). Consequently, the entropy identity results in

(119) $$\begin{array}{cc} S^{Ak}{}_{;k}\;\equiv & {\left(\;s^{Ak}-\lambda^{A}q^{Ak}-\lambda_{l}^{A}t^{Akl}-\Lambda_{m}^{A}\Xi^{Akm}-\Lambda_{mn}^{A}s^{Akmn}-\kappa^{A}J^{Am}h_{m}^{Ak}\right)}_{;k}\\ & +\kappa^{A}{}^{\left(ex\right)}\Gamma^{A}+\left(\lambda^{A}u_{l}^{A}+\lambda_{l}^{A}\right)k^{Al}+\left(\Lambda_{b}^{A}u_{a}^{A}+\Lambda_{ab}^{A}\right)\frac{1}{c^{2}}m^{Aab}+\\ & +\kappa^{A}{}_{;k}J^{Am}h_{m}^{Ak}+\kappa^{A}\left[{}^{\left(in\right)}\;\Gamma^{A}+{\left(J^{Am}h_{m}^{Ak}\right)}_{;k}\right]+\\ & +\lambda {}^{A}{}_{;k}q^{Ak}+\;\lambda^{A}u^{A}{}_{l;k}\left(\pi^{Akl}+u^{Ak}p^{Al}\right)+\\ & +\;\lambda^{A}{}_{l;k}\left(\frac{1}{c^{2}}e^{A}u^{Ak}u^{Al}+\frac{1}{c^{2}}q^{Ak}u^{Al}+t^{Akl}\right)+\\ & +\Lambda_{b;k}^{A}\Xi^{Akb}-\Lambda_{b}^{A}u_{a}^{A}\left(s^{Akab}{}_{;k}+\frac{1}{c^{2}}\overset{•}{s}\;{}^{Aab}\right)+\\ & +\overset{•}{\Lambda }\;{}_{ab}^{A}\frac{1}{c^{4}}u^{A[a}\Xi^{Ab]}+\Lambda {}_{ab;k}^{A}\left(\frac{1}{c^{2}}u^{A[a}\Xi^{Akb]}+s^{Akab}\right)\;=\\ = & \sigma^{A}+\varphi^{A}. \end{array}$$

We now specify the entropy flux density $s^{Ak}$ and the entropy supply $\varphi^{A}$ in the next section.

#### 6.2.2. Entropy Flux, -Supply and -Production

According to the first row of Equation (119), we define the *entropy flux density*

(120) $$\begin{array}{c} \mathrm{Setting}V:\;\\ s^{Ak}\;\overset{•}{=}\;\lambda^{A}q^{Ak}+\lambda_{l}^{A}t^{Akl}+\Lambda_{m}^{A}\Xi^{Akm}+\Lambda_{mn}^{A}s^{Akmn}+\kappa^{A}J^{Am}h_{m}^{Ak}. \end{array}$$

We now split the entropy identity in Equation (119) into the entropy production and the entropy supply. For this end, we need a criterion to distinguish between entropy production and supply. Such a criterion is clear for discrete systems: a local isolation suppresses the entropy supply but not the entropy production. Isolation means: the second row in Equation (119) vanishes, if the ${}^{A}$-component is isolated from the exterior of the mixture. Consequently, we define the *entropy supply* as follows

(121) $$\begin{array}{c} \mathrm{Setting}\mathrm{VI}:\;\\ \varphi^{A}\;\overset{•}{=}\;\kappa^{A}{}^{\left(ex\right)}\Gamma^{A}+\left(\lambda^{A}u_{l}^{A}+\lambda_{l}^{A}\right)k^{Al}+\left(\Lambda_{b}^{A}u_{a}^{A}+\Lambda_{ab}^{A}\right)\frac{1}{c^{2}}m^{Aab}, \end{array}$$

with the result that the entropy identity in Equation (119) transfers into the entropy production density by taking Equations (120) and (121) into account

(122) $$\begin{array}{cc} \sigma^{A}\;=\;\; & +\kappa^{A}{}_{;k}J^{Am}h_{m}^{Ak}+\kappa^{A}\left[{}^{\left(in\right)}\;\Gamma^{A}+\left(J^{Am}h_{m}^{Ak}\right){}_{;k}\right]+\\ & +\lambda {}^{A}{}_{;k}q^{Ak}+\;\lambda^{A}u^{A}{}_{l;k}\left(\pi^{Akl}+u^{Ak}p^{Al}\right)+\\ & +\;\lambda^{A}{}_{l;k}\left(\frac{1}{c^{2}}e^{A}u^{Ak}u^{Al}+\frac{1}{c^{2}}q^{Ak}u^{Al}+t^{Akl}\right)+\\ & +\Lambda_{b;k}^{A}\Xi^{Akb}-\Lambda_{b}^{A}u_{a}^{A}\left(s^{Akab}{}_{;k}+\frac{1}{c^{2}}\overset{•}{s}\;{}^{Aab}\right)+\\ & +\overset{•}{\Lambda }\;{}_{ab}^{A}\frac{1}{c^{4}}u^{A[a}\Xi^{Ab]}+\Lambda {}_{ab;k}^{A}\left(\frac{1}{c^{2}}u^{A[a}\Xi^{Akb]}+s^{Akab}\right). \end{array}$$

As expected, the entropy production is composed of terms which are a product of “forces” and “fluxes” as in the non-relativistic case (the mass production ${}^{\left(in\right)}\Gamma^{A}$ due to chemical reactions can be expressed by the time rate of the reaction velocity; see Equation (A19) in Appendix A.3). The expressions $s^{A},\;s^{Ak},\;\varphi^{A}$ and $\sigma^{A}$ contain Lagrange multipliers, which are introduced for formulating the entropy identity in Equation (89) playing up to here the role of place-holders. Their physical meaning is discussed in the next section.

### 6.3. Fields of Lagrange Multipliers

From non-relativistic physics, we know the physical dimensions of the entropy density and the entropy flux density by taking Equations (36) and (38) into account

(123) $$\left[s^{A}\right]\;=\;\left[e^{A}\right]\frac{1}{K}\;=\;\frac{Nm}{m^{3}}\frac{1}{K},\;\left[s^{Ak}\right]\;=\;\left[q^{Ak}\right]\frac{1}{K}\;=\;\frac{Nm}{m^{3}}\frac{m}{s}\frac{1}{K}.$$

According to Equation (111), we have the following equation of physical dimensions

(124) $$\left[s^{A}\right]\;=\;\left[\lambda^{A}\right]\left[e^{A}\right].$$

Taking Equations (123)${}_{1}$ and (36) into account, we obtain

(125) $$\frac{N}{m^{2}}\frac{1}{K}\;=\;\left[\lambda^{A}\right]\frac{N}{m^{2}}\;⟶\;\left[\lambda^{A}\right]\;=\;\frac{1}{K},$$

which means $\lambda^{A}$ is a reciprocal temperature belonging to the ${}^{A}$-component. Therefore, we accept the following

(126) $$\begin{array}{c} \mathrm{Setting}\mathrm{VII}:\;\\ \lambda^{A}\;\overset{•}{=}\;\frac{\nu^{A}}{\Theta^{A}},\; \end{array}$$

with the partial temperature $\Theta^{A}$ of the ${}^{A}$-component (this temperature is a non-equilibrium one, the contact temperature [22,23,24] which should not be confused with the thermostatic equilibrium temperature $\Theta_{eq}^{A}=T,\;\forall A$) and a scalar $\nu^{A}$, which is suitably chosen below.

According to Equation (120), we have the following equation of physical dimensions

(127) $$\left[s^{Ak}\right]\;=\;\left[\kappa^{A}\right]\left[J^{Am}\right]\left[h_{m}^{Ak}\right].$$

Taking Equations (123)${}_{2}$, (15)${}_{2}$ and (35)${}_{1}$ into account, we obtain

(128) $$\frac{N}{ms}\frac{1}{K}\;=\;\left[\kappa^{A}\right]\frac{kg}{m^{3}}\frac{m}{s}1\;⟶\;\left[\kappa^{A}\right]\;=\;\frac{m^{2}}{s^{2}}\frac{1}{K}.$$

We know from the non-relativistic Gibbs equation that the chemical potentials $\mu^{A}$ have the physical dimension of the specific energy $e^{A}/\varrho^{A}$

(129) $$\left[\mu^{A}\right]\;=\frac{\left[e^{A}\right]}{\left[\varrho^{A}\right]}\;=\;\frac{N}{m^{2}}\frac{m^{3}}{kg}\;=\;\frac{kg\;m}{s^{2}}\frac{m}{kg}\;=\;\frac{m^{2}}{s^{2}}\;=\;K\left[\kappa^{A}\right].$$

Consequently, we choose by taking Equation (129) into consideration

(130) $$\begin{array}{c} \mathrm{Setting}\mathrm{VIII}:\;\\ \kappa^{A}\;\overset{•}{=}\;\frac{\mu^{A}}{\Theta^{A}}.\; \end{array}$$

According to the second term of Equation (121), we have the following equation of physical dimensions

(131) $$\left[\lambda^{Ak}\right]\;=\;\left[\lambda u^{Ak}\right]\;=\;\frac{1}{K}\frac{m}{s},$$

which means $\lambda^{Ak}$ is proportional to a velocity and simultaneous perpendicular to $u^{Ak}$ according to Equation (89)${}_{1}$. Consequently, only the velocity $u^{m}$ of the mixture remains for defining $\lambda^{Ak}$ in accordance with Equation (89)${}_{1}$

(132) $$\begin{array}{c} \mathrm{Setting}\mathrm{IX}:\;\\ \lambda^{Ak}\;\overset{•}{=}\;\frac{1}{\Theta^{A}}u^{m}h_{m}^{Ak}.\; \end{array}$$

We know from the non-relativistic continuum theory and Equations (25)${}_{1}$ and (36) the following connection of the physical dimensions (angular momentum = spin density per time)

(133) $$\left[k_{l}^{A}\right]m\;=\;\frac{N}{m^{3}}m\;=\;\left[m^{ab}\right]\;=\;\frac{1}{s}\left[s^{ab}\right]\;=\;\frac{N}{m^{2}}.$$

From the last term of Equation (74) follows by taking Equation (133) into account

(134) $$\left[S^{Akab}\right]\;=\;\frac{s}{m}\left[s^{Aab}\right]\;=\;\frac{s^{2}}{m^{3}}N.$$

From the first term of the third row of Equation (91) follows by use of Equations (86)${}_{1}$ and (123)${}_{4}$

(135) $$\left[S^{Ak}\right]\;=\;\left[\Lambda_{a}^{A}\right]\frac{m}{s}\left[S^{Akab}\right]\;=\;\left[\Lambda_{ab}^{A}\right]\left[S^{Akab}\right]\;=\;\frac{Nm}{m^{3}}\frac{m}{s}\frac{1}{K},$$

and taking Equation (134) into account, we obtain

(136) $$\left[\Lambda_{a}^{A}\right]\;=\;\frac{1}{K}\frac{m}{s^{2}},\;\left[\Lambda_{ab}^{A}\right]\;=\;\frac{1}{K}\frac{m}{s^{2}}\frac{m}{s}.$$

In accordance with Equation (89)${}_{2,3}$ and analogously to Equation (132), the relations in Equation (136) allow the following

(137) $$\begin{array}{c} \mathrm{Setting}X:\;\\ \Lambda_{a}^{A}\;\overset{•}{=}\;\frac{\overset{•}{u}\;{}_{a}^{A}}{\Theta^{A}},\;\Lambda_{ab}^{A}\;\overset{•}{=}\;\frac{1}{\Theta^{A}}\overset{•}{u}\;{}_{[m}^{A}u_{n]}h_{a}^{Am}h_{b}^{An}\;=\;\frac{1}{2\Theta^{A}}\left(\overset{•}{u}{\;}_{m}^{A}u_{n}-\overset{•}{u}{\;}_{n}^{A}u_{m}\right)h_{a}^{Am}h_{b}^{An}. \end{array}$$

Inserting the Lagrange multipliers into the expression of entropy density in Equation (111), of entropy flux density in Equation (120) and of entropy supply in Equation (121), we obtain by use of Equation (72)

(138) $$\begin{array}{ccc} s^{A} & = & \frac{1}{\Theta^{A}}\left(\mu^{A}\varrho^{A}+\nu^{A}\left(e^{A}+p^{A}\right)+u_{m}p^{Am}+\overset{•}{u}\;{}_{m}^{A}\frac{1}{c^{2}}\Xi^{Am}+\overset{•}{u}\;{}_{[a}^{A}u_{b]}\frac{1}{c^{2}}s^{Aab}\right), \end{array}$$

(139) $$\begin{array}{ccc} s^{Ak} & = & \frac{1}{\Theta^{A}}\left(\nu^{A}q^{Ak}+u_{m}t^{Akm}+\mu^{A}J^{Am}h_{m}^{Ak}+\overset{•}{u}\;{}_{m}^{A}\Xi^{Akm}+\overset{•}{u}\;{}_{[a}^{A}u_{b]}s^{Akab}\right),\\ \varphi^{A} & = & \frac{1}{\Theta^{A}}\{\mu^{A}{}^{\left(ex\right)}\Gamma^{A}+\left(\nu^{A}u_{l}^{A}+u_{m}h_{l}^{Am}\right)k^{Al}+ \end{array}$$

(140) $$\begin{array}{c} +\left(\overset{•}{u}{}_{b}^{A}u_{a}^{A}+\overset{•}{u}\;{}_{[m}^{A}u_{n]}h_{a}^{Am}h_{b}^{An}\right)\frac{1}{c^{2}}m^{Aab}\},\; \end{array}$$

The entropy production density in Equation (118) results by inserting the Lagrange multipliers in Equations (126), (130), (132) and (137)

(141) $$\begin{array}{cc} \sigma^{A}\;=\;\; & {\left(\frac{\mu^{A}}{\Theta^{A}}\right)}_{;k}J^{Am}h_{m}^{Ak}+\frac{\mu^{A}}{\Theta^{A}}\left[{}^{\left(in\right)}\;\Gamma^{A}+\left(J^{Am}h_{m}^{Ak}\right){}_{;k}\right]+\\ & +{\left(\frac{\nu^{A}}{\Theta^{A}}\right)}_{;k}q^{Ak}+\frac{\nu^{A}}{\Theta^{A}}u^{A}{}_{l;k}\left(\pi^{Akl}+u^{Ak}p^{Al}\right)+\\ & +\;{\left(\frac{1}{\Theta^{A}}u^{m}h_{ml}^{A}\right)}_{;k}\left(\frac{1}{c^{2}}e^{A}u^{Ak}u^{Al}+\frac{1}{c^{2}}q^{Ak}u^{Al}+t^{Akl}\right)+\\ & +{\left(\frac{\overset{•}{u}\;{}_{b}^{A}}{\Theta^{A}}\right)}_{;k}\Xi^{Akb}-\underset{︸}{\frac{\overset{•}{u}\;{}_{b}^{A}}{\Theta^{A}}u_{a}^{A}}\left(s^{Akab}{}_{;k}+\underset{︸}{\frac{1}{c^{2}}\overset{•}{s}\;{}^{Aab}}\right)+\\ & +\left(\frac{1}{\Theta^{A}}\overset{•}{u}\;{}_{[m}^{A}u_{n]}h_{a}^{Am}h_{b}^{An}\right){}^{•}\frac{1}{c^{4}}u^{A[a}\Xi^{Ab]}+\\ & +{\left(\frac{1}{\Theta^{A}}\overset{•}{u}\;{}_{[m}^{A}u_{n]}h_{a}^{Am}h_{b}^{An}\right)}_{;k}\left(\frac{1}{c^{2}}u^{A[a}\Xi^{Akb]}+s^{Akab}\right). \end{array}$$

The underbraced terms result in

(142) $$\overset{•}{u}\;{}_{b}^{A}u_{a}^{A}\overset{•}{s}\;{}^{Aab}\;=\;\overset{•}{u}\;{}_{b}^{A}{\underset{=0}{\underset{︸}{\left(u_{a}^{A}s^{Aab}\right)}}}^{{}^{•}}-\overset{•}{u}\;{}_{[b}^{A}\overset{•}{u}\;{}_{a]}^{A}s^{Aab}\;=\;0,$$

which means the spin density does not appear in the entropy production.

The first four terms of the entropy production describe the four classical reasons of irreversibility: diffusion, chemical reactions, heat conduction and internal friction with, by the momentum flux, density modified non-equilibrium viscous tensor. The fifth term of Equation (141) (which vanishes in equilibrium and for free 1-component systems, as we show below) is typical for an interacting ${}^{A}$-component as a part of the mixture because it contains the 4-velocity of the mixture $u^{m}$. The same is true for the last two spin terms which vanish for 1-component systems. In any case, all spin terms of the fields in Equations (138)–(141) related to entropy vanish with the 4-acceleration.

Up to now, a further phenomenon of irreversibility was not taken into consideration: the multi-temperature relaxation which is discussed in the next section.

### 6.4. Multi-Temperature Relaxation and the Partial Temperatures

Because the different components of the mixture have different partial (reciprocal) temperatures $\lambda^{A},\;A=1,2,\ldots ,Z$, a multi-temperature relaxation (do not take the multi-temperature relaxation for the heat conduction which is caused by a temperature gradient in contrast to the multi-temperature relaxation) takes place, which is an irreversible phenomenon. Consequently, the multi-temperature relaxation has to be taken into account by adding a suitable zero to the entropy identity as done in Equation (89).

A heat transfer $H^{AB}$ between two components of the mixture—*A* and *B*—takes place by multi-temperature relaxation, if the corresponding temperatures of the components are different from each other. Consequently, the entropy exchange between these two components is

(143) $$\begin{array}{c} \mathrm{Setting}\mathrm{XI}:\;\\ G^{AB}\;:=\;H^{AB}\left(\frac{1}{\Theta^{A}}-\frac{1}{\Theta^{B}}\right),\;H^{AB}=-H^{BA},\;\overset{•}{H}\;{}^{AB}=0,. \end{array}$$

Here, $H^{AB}$ is an energy density and $G^{AB}$ an entropy density according to Equation (124)

(144) $$\left[H^{AB}\right]\;=\;\left[e^{A}\right],\;\;\;\left[G^{AB}\right]\;=\;\left[e^{A}\right]\frac{1}{K}\;=\;\left[s^{A}\right].$$

For the ${}^{A}$-component, this results according to Equation (143)${}_{2}$ in

(145) $$\begin{array}{ccc} H^{A} & := & \sum_{B}H^{AB},\;\sum_{AB}H^{AB}\;=\;0,\;\sum_{A}H^{A}\;=\;0, \end{array}$$

(146) $$\begin{array}{ccc} G^{A} & := & \sum_{B}H^{AB}\left(\frac{1}{\Theta^{A}}-\frac{1}{\Theta^{B}}\right), \end{array}$$

(147) $$\begin{array}{ccc} \sum_{A}G^{A} & = & \sum_{AB}H^{AB}\left(\frac{1}{\Theta^{A}}-\frac{1}{\Theta^{B}}\right)\;\neq \;0,\;\mathrm{if}\;\Theta^{A}\neq \Theta^{B}. \end{array}$$

The entropy exchange of the ${}^{A}$-component according to the multi-temperature exchange in Equations (146)${}_{1}$ and (143)${}_{3}$ has now to be introduced into the entropy identity in Equation (110). Because $G^{A}$ has the same physical dimension as $s^{A}$, according to Equation (144)${}_{2}$, it fits into the first row of Equation (110). Therefore, we add the zero

(148) $$0\;=\;-{\left[u^{Ak}G^{A}\right]}_{;k}+\sum_{B}{\left[u^{Ak}H^{AB}\left(\frac{1}{\Theta^{A}}-\frac{1}{\Theta^{B}}\right)\right]}_{;k}$$

to Equation (110). Taking Equation (143)${}_{3}$ into account and inserting

(149) $$\sum_{B}{\left[u^{Ak}H^{AB}\left(\frac{1}{\Theta^{A}}-\frac{1}{\Theta^{B}}\right)\right]}_{;k}\;=\;u^{Ak}{}_{;k}G^{A}+\sum_{B}H^{AB}{\left(\frac{1}{\Theta^{A}}-\frac{1}{\Theta^{B}}\right)}^{{}^{•}},$$

into Equation (148)

(150) $$0\;=\;-{\left[u^{Ak}G^{A}\right]}_{;k}+\;u^{Ak}{}_{;k}G^{A}+\sum_{B}H^{AB}{\left(\frac{1}{\Theta^{A}}-\frac{1}{\Theta^{B}}\right)}^{{}^{•}},$$

we obtain three additional terms which can be directly introduced into the entropy identity without defining an additional Lagrange multiplier. According to Equation (110), the three terms of Equation (150) are attached as follows

(151) $$\begin{array}{ccc} -G^{A} & ⟶ & \mathrm{entropy}\;\mathrm{density} \end{array}$$

(152) $$\begin{array}{ccc} u^{Ak}{}_{;k}G^{A} & ⟶ & \mathrm{entropy}\;\mathrm{supply}, \end{array}$$

(153) $$\begin{array}{ccc} \sum_{B}H^{AB}{\left(\frac{1}{\Theta^{A}}-\frac{1}{\Theta^{B}}\right)}^{{}^{•}} & ⟶ & \mathrm{entropy}\;\mathrm{production}\;\mathrm{density}, \end{array}$$

Introducing these terms as demonstrated in Section 6.1 into the entropy identity in Equation (119), the entropy density in Equation (138) becomes

(154) $$s^{A}\;=\;\frac{1}{\Theta^{A}}\left(\mu^{A}\varrho^{A}+\nu^{A}\left(e^{A}+p^{A}\right)+u_{m}p^{Am}+\overset{•}{u}\;{}_{m}^{A}\frac{1}{c^{2}}\Xi^{Am}+\overset{•}{u}\;{}_{[a}^{A}u_{b]}\frac{1}{c^{2}}s^{Aab}\right)+G^{A},$$

and the state space in Equation (113) is extended by $G^{A}/\varrho$

(155) $$z^{A}\;=\;\left(c^{A},\frac{1}{\varrho },\frac{e^{A}}{\varrho },\frac{p^{Al}}{\varrho },\frac{\Xi^{Am}}{\varrho },\frac{s^{Amn}}{\varrho },\frac{G^{A}}{\varrho }\right),\;c^{A}\;:=\;\frac{\varrho^{A}}{\varrho },$$

and consequently the Gibbs equation (114) by ${\left(G^{A}/\varrho \right)}^{{}^{•}}$. The Gibbs–Duhem equation (Equation (115)) is untouched by including the multi-temperature relaxation.

According to Equation (152), the entropy supply in Equation (140) results in

(156) $$\begin{array}{c} \varphi^{A}\;=\;\frac{1}{\Theta^{A}}\{\mu^{A}{}^{\left(ex\right)}\Gamma^{A}+\left(\nu^{A}u_{l}^{A}+u_{m}h_{l}^{Am}\right)k^{Al}+\;\\ +\left(\overset{•}{u}{}_{b}^{A}u_{a}^{A}+\overset{•}{u}\;{}_{[m}^{A}u_{n]}h_{a}^{Am}h_{b}^{An}\right)\frac{1}{c^{2}}m^{Aab}\}+u^{Ak}{}_{;k}G^{A}, \end{array}$$

and the entropy production density in Equation (141) becomes by Equation (153)

(157) $$\begin{array}{ccc} \sigma^{A}\; & = & {\left(\frac{\mu^{A}}{\Theta^{A}}\right)}_{;k}J^{Am}h_{m}^{Ak}+\frac{\mu^{A}}{\Theta^{A}}\left[{}^{\left(in\right)}\;\Gamma^{A}+\left(J^{Am}h_{m}^{Ak}\right){}_{;k}\right]+\\ & & +{\left(\frac{\nu^{A}}{\Theta^{A}}\right)}_{;k}q^{Ak}+\frac{\nu^{A}}{\Theta^{A}}u^{A}{}_{l;k}\left(\pi^{Akl}+u^{Ak}p^{Al}\right)+\\ & & +\;{\left(\frac{1}{\Theta^{A}}u^{m}h_{ml}^{A}\right)}_{;k}\left(\frac{1}{c^{2}}e^{A}u^{Ak}u^{Al}+\frac{1}{c^{2}}q^{Ak}u^{Al}+t^{Akl}\right)+\\ & & +\sum_{B}H^{AB}{\left(\frac{1}{\Theta^{A}}-\frac{1}{\Theta^{B}}\right)}^{{}^{•}}+\\ & & +{\left(\frac{\overset{•}{u}\;{}_{b}^{A}}{\Theta^{A}}\right)}_{;k}\Xi^{Akb}-\frac{\overset{•}{u}\;{}_{b}^{A}}{\Theta^{A}}u_{a}^{A}s^{Akab}{}_{;k}+\\ & & +\left(\frac{1}{\Theta^{A}}\overset{•}{u}\;{}_{[m}^{A}u_{n]}h_{a}^{Am}h_{b}^{An}\right){}^{•}\frac{1}{c^{4}}u^{A[a}\Xi^{Ab]}+\\ & & +{\left(\frac{1}{\Theta^{A}}\overset{•}{u}\;{}_{[m}^{A}u_{n]}h_{a}^{Am}h_{b}^{An}\right)}_{;k}\left(\frac{1}{c^{2}}u^{A[a}\Xi^{Akb]}+s^{Akab}\right). \end{array}$$

The ten terms of the entropy production density in Equation (157) have—as already discussed after Equation (141)—the following meaning:

- diffusion: $\left(\mu^{A}/\Theta^{A}\right){}_{;k}J^{Am}h^{Ak_{m}}$;
- by diffusion modified chemical reaction: $\left(\mu^{A}/\Theta^{A}\right)\left({}^{\left(in\right)}\Gamma^{A}+{\left(J^{Am}h_{m}^{Ak}\right)}_{;k}\right)$;
- heat conduction: $\left(\nu^{A}/\Theta^{A}\right){}_{;k}q^{Ak}$;
- multi-component modified internal friction: $\left(\nu^{A}/\Theta^{A}\right)u^{A}{}_{l,k}\left(\pi^{Akl}+u^{Ak}p^{Al}\right)$;
- multi-component interaction (this term vanishes in equilibrium and for 1-component systems: see Section 7): $\left(u^{m}h_{ml}^{A}/\Theta^{A}\right){}_{;k}\left(\frac{1}{c^{2}}e^{A}u^{Ak}u^{Al}+\frac{1}{c^{2}}q^{Ak}u^{Al}+t^{Akl}\right)$;
- multi-temperature relaxation: $\sum_{B}H^{AB}{\left(\left(1/\Theta^{A}\right)-\left(1/\Theta^{B}\right)\right)}^{{}^{•}}$; and
- four terms describing entropy production by the spin $S^{Akab}$.

### 6.5. The 4-Entropy

We need the 4-entropy of the ${}^{A}$-component for describing thermodynamics of a mixture. Starting with Equations (86)${}_{1}$, (154) and (139), we obtain

(158) $$\begin{array}{c} S^{Ak}=\left\{\frac{1}{\Theta^{A}}\left(\mu^{A}\varrho^{A}+\nu^{A}\left(e^{A}+p^{A}\right)+u_{m}p^{Am}+\overset{•}{u}\;{}_{m}^{A}\frac{1}{c^{2}}\Xi^{Am}+\overset{•}{u}\;{}_{[a}^{A}u_{b]}\frac{1}{c^{2}}s^{Aab}\right)+G^{A}\right\}u^{Ak}+\\ +\frac{1}{\Theta^{A}}\left(\nu^{A}q^{Ak}+u_{m}t^{Akm}+\mu^{A}J^{Am}h_{m}^{Ak}+\overset{•}{u}\;{}_{m}^{A}\Xi^{Akm}+\overset{•}{u}\;{}_{[a}^{A}u_{b]}s^{Akab}\right).\; \end{array}$$

Rearranging results in

(159) $$\begin{array}{ccc} S^{Ak} & = & \frac{\mu^{A}}{\Theta^{A}}\left(N^{Ak}+J^{Am}h_{m}^{Ak}\right)+\\ & & +\frac{1}{\Theta^{A}}\left\{\nu^{A}\left(\left(e^{A}+p^{A}\right)u^{Ak}+q^{Ak}\right)+u_{m}\left(u^{Ak}p^{Am}+t^{Akm}\right)\right\}+\\ & & +\frac{1}{\Theta^{A}}\overset{•}{u}\;{}_{m}^{A}\left(\frac{1}{c^{2}}\Xi^{Am}u^{Ak}+\Xi^{Akm}\right)+\frac{1}{\Theta^{A}}\overset{•}{u}\;{}_{[a}^{A}u_{b]}\left(\frac{1}{c^{2}}s^{Aab}u^{Ak}+s^{Akab}\right)+G^{A}u^{Ak}.\; \end{array}$$

The transition from the interacting ${}^{A}$-component to the free 1-component system is considered in Section 7 and that to the mixture in Section 8. All quantities introduced up to here are non-equilibrium ones, because we do not consider equilibrium conditions up to now. This is done in the next section.

### 6.6. Equilibrium

#### 6.6.1. Equilibrium Conditions

Equilibrium is defined by *equilibrium conditions* which are divided into *basic* and *supplementary* ones [11,12]. The basic equilibrium conditions are given by vanishing entropy production, vanishing entropy flux density and vanishing entropy supply (the sign ≐ stands for a setting which implements an equilibrium condition):

(160) $$\sigma_{eq}^{A}\;≐\;0\;\wedge \;s_{eq}^{Ak}\;≐\;0\;\wedge \;\varphi_{eq}^{A}\;≐\;0.$$

A first supplementary equilibrium condition is the vanishing of all diffusion flux densities. According to Equation (15)${}_{1}$, we obtain

(161) $$J_{k}^{Aeq}\;≐\;0\;⟶\;u_{k}^{Aeq}\;=\;f_{eq}^{A}u_{k}^{eq}\;⟶\;c^{2}\;=\;\;f_{eq}^{A}u_{k}^{eq}u_{eq}^{Ak}.$$

Taking Equation (9)${}_{1}$ into account, Equation (161)${}_{3}$ results in

(162) $${\left(f_{eq}^{A}\right)}^{2}\;=\;1\;⟶\;f_{eq}^{A}\;=\;\pm 1.$$

Consequently, we have to demand beyond Equation (161)${}_{1}$ the supplementary equilibrium condition that the mass densities are additive in equilibrium. We obtain according to Equations (9)${}_{2}$ and (17)${}_{2}$

(163) $$\varrho_{eq}\;≐\;\sum_{A}\varrho_{eq}^{A}\;⟶\;f_{eq}^{A}\;=\;1\;⟶\;w_{eq}^{A}\;=\;0.$$

Taking Equations (161)${}_{2}$, (132) and (137)${}_{2}$ into account, Equation (163)${}_{2}$ yields

(164) $$u_{k}^{Aeq}\;=\;u_{k}^{eq}\;⟶\;\lambda {}_{eq}^{Ak}\;=\;0\;\wedge \;g_{eq}^{Am}\;=\;0\;\wedge \;\Lambda_{ab}^{Aeq}\;=\;0.$$

Further supplementary equilibrium conditions are given by vanishing covariant time derivatives, except that of the four-velocity:

(165) $${⊞}_{eq}^{•}\;≐\;0,\;⊞\;\neq \;u^{l},$$

that means $\overset{•}{u}{}_{eq}^{l}$ is in general not zero in equilibrium. Consequently, the time derivatives of all expressions which contain the 4-velocity must be calculated separately, as we show below.

According to Equation (165)${}_{1}$, we obtain

(166) $$\overset{•}{\varrho }\;{}_{eq}^{A}\;=\;0,\;{\left(\frac{\nu^{A}}{\Theta^{A}}\right)}_{eq}^{{}^{•}}\;=\;0,$$

and the (3+1)-components of the energy–momentum tensor, Equations (29) and (30), satisfy

(167) $$\overset{•}{e}{}_{eq}^{A}=0,\;\overset{•}{p}{}_{eq}^{Al}=0,\;\overset{•}{q}{}_{eq}^{Ak}=0,\;\overset{•}{p}{}_{eq}^{A}=0,\;\overset{•}{\pi }{}_{eq}^{Akl}=0.$$

Starting with Equation (9)${}_{1}$, we have

(168) $$\overset{•}{f}\;{}_{eq}^{A}\;=\;\frac{1}{c^{2}}\left(\overset{•}{u}{}_{m}^{Aeq}u_{eq}^{m}+u{}_{m}^{Aeq}\overset{•}{u}\;{}_{eq}^{m}\right).$$

Taking Equation (164)${}_{1}$ into account, this results in

(169) $$\overset{•}{f}\;{}_{eq}^{A}\;=\;0\;⟶\;\overset{•}{w}\;{}_{eq}^{A}\;=\;0.\;$$

In equilibrium, we have according to Equations (164)${}_{1}$ and (19)

(170) $$h_{leq}^{Am}\;=\;h_{leq}^{m},$$

and according to Equation (132) resulting in

(171) $$\lambda^{Aeq}{}_{l;k}\;=\;{\left(\frac{1}{\Theta^{A}}u_{m}h_{l}^{Am}\right)}_{;k}^{eq}\;=\;0.$$

Despite $\Lambda_{a}^{Aeq}\neq 0$ according to Equations (137)${}_{1}$ and (165)${}_{2}$, the time derivatives of the Lagrange multipliers vanish in equilibrium, and, according to Equations (114) and (115), Gibbs and Gibbs–Duhem equations are identically satisfied in equilibrium, if the “shift of the time derivative” is performed on the fifth term of Equation (115)

(172) $$\begin{array}{c} \overset{•}{\Lambda }{}_{m}^{A}\frac{\Xi^{Am}}{\varrho }\;=\;{\left[\frac{\overset{•}{u}{}_{m}^{A}}{\Theta^{A}}\frac{\Xi^{Am}}{\varrho }\right]}^{{}^{•}}-\Lambda {}_{m}^{A}{\left(\frac{\Xi^{Am}}{\varrho }\right)}^{{}^{•}}\;=\;\\ =\;{\left[{\left(\underset{=0}{\underset{︸}{\frac{u{}_{m}^{A}}{\Theta^{A}}\frac{\Xi^{Am}}{\varrho }}}\right)}^{{}^{•}}-u_{m}^{A}{\left(\frac{\Xi^{Am}}{\Theta^{A}\varrho }\right)}^{{}^{•}}\right]}^{{}^{•}}-\Lambda {}_{m}^{A}{\left(\frac{\Xi^{Am}}{\varrho }\right)}^{{}^{•}}\;\overset{eq}{⟶}\;0. \end{array}$$

Another supplementary equilibrium condition is the vanishing of the mass production terms in Equation (13)${}_{3,4}$

(173) $${}^{\left(ex\right)}\Gamma_{eq}^{A}\;≐\;0\;\wedge \;{}^{\left(in\right)}\Gamma_{eq}^{A}\;≐\;0\;⟶\;\Gamma_{eq}^{A}\;=\;0$$

Thus, we obtain from Equations (1)${}_{2}$, (166)${}_{1}$ and (173)${}_{3}$

(174) $$\varrho^{A}{}_{;k}u^{Ak}+\varrho^{A}u^{Ak}{}_{;k}\;=\;\Gamma^{A}\;⟶\;u_{eq}^{Ak}{}_{;k}\;=\;0.$$

The equilibrium temperature is characterized by vanishing multi-temperature relaxation

(175) $$\Theta_{eq}^{A}\;≐\;\Theta_{eq}^{B}\;≐\;\Theta_{eq}^{C}\;≐\ldots \;=:\;\Theta_{eq}⟶G^{A}\;=\;0,\;\forall A.$$

Often one can find in the literature [25] the case of equilibrium of multi-temperature relaxation: although out of equilibrium, only one temperature is considered in multi-component systems. This case is realistic, if the relaxation of multi-temperature relaxation to equilibrium is remarkably faster than that of the other non-equilibrium variables [26].

Taking Equations (164)${}_{1}$, (29)${}_{2}$ and (175) into account, the entropy density in Equation (154) becomes in equilibrium using the shift of the time derivative

(176) $$s_{eq}^{A}\;=\;\frac{1}{\Theta_{eq}^{A}}\left(\mu_{eq}^{A}\varrho_{eq}^{A}+\nu_{eq}^{A}\left(e_{eq}^{A}+p_{eq}^{A}\right)\right).$$

Beyond the usual expression of the entropy density in thermostatics (below, we show that $\nu_{eq}^{A}=1$), it includes an acceleration dependent spin term. The energy density and the pressure are here defined by the (3+1)-decomposition in Equation (28) of the energy–momentum tensor. The chemical potential is as well as the temperature introduced as a Lagrange multiplier.

Taking Equations (164)${}_{1}$, (161)${}_{1}$ and (175) into account, the entropy flux density in Equation (139) vanishes in equilibrium, resulting in

(177) $$0\;=\;\nu^{A}q_{eq}^{Ak}+\overset{•}{u}\;{}_{m}^{Aeq}\Xi_{eq}^{Akm}\;⟶\;q_{eq}^{Ak}\;=\;0,$$

using the shift of the time derivative according to Equations (172) and (165).

Finally, the entropy supply in Equation (156) results in

(178) $$0\;=\;\nu_{eq}^{A}u_{l}^{Aeq}k_{eq}^{Al}+\overset{•}{u}\;{}_{b}^{Aeq}u_{a}^{Aeq}\frac{1}{c^{2}}m_{eq}^{Aab},$$

which means the power exchange caused by the force density and by the angular momentum density vanishes in equilibrium.

The entropy production in Equation (157) has to vanish in equilibrium according to the basic equilibrium condition in Equation (160)${}_{1}$. Taking Equations (173)${}_{2}$, (161)${}_{1}$, (177), (167)${}_{2}$, (164)${}_{1}$ and (175) into account and using Equations (165) and (157) results in

(179) $$0\;=\;\frac{\nu_{eq}^{A}}{\Theta_{eq}^{A}}u^{Aeq}{}_{l;k}\pi_{eq}^{Akl}+{\left(\frac{\overset{•}{u}\;{}_{b}^{A}}{\Theta^{A}}\right)}_{;k}^{eq}\Xi_{eq}^{Akb}-\frac{\overset{•}{u}\;{}_{b}^{Aeq}}{\Theta_{eq}}u_{a}^{Aeq}{\left(s^{Akab}{}_{;k}\right)}^{eq}.$$

The third term of the second row of Equation (157) vanishes by shift of the time derivative. In equilibrium, spin terms appear in the vanishing power exchange in Equation (178) and in the spin modified internal friction in Equation (179).

As demonstrated, equilibrium of an ${}^{A}$-component in the mixture is described by three basic equilibrium conditions in Equation (160) and six supplementary ones: Equations (161)${}_{1}$, (163)${}_{1}$, (165), (173)${}_{1,2}$ and (175). Often, we can find in the literature [27,28] equilibrium conditions which are different from those postulated here. The reason is that entropy production and supply and the entropy flux as starting points for the basic equilibrium conditions differ from the expressions in Equations (138)–(141). Such different equilibrium conditions and their derivations are considered in the next two sections.

#### 6.6.2. Killing Relation of the 4-Temperature

Starting with Equation (93)${}_{1}$, we now consider the following relations

(180) $$\begin{array}{ccc} \left(\lambda^{A}{}_{;k}u_{l}^{A}+\lambda^{A}u_{l;k}^{A}\right)\frac{1}{c^{2}}e^{A}u^{Ak}u^{Al} & = & \overset{•}{\lambda }\;{}^{A}e^{A}, \end{array}$$

(181) $$\begin{array}{ccc} \left(\lambda^{A}{}_{;k}u_{l}^{A}+\lambda^{A}u_{l;k}^{A}\right)u^{Ak}p^{Al} & = & -\lambda^{A}u_{l}^{A}\overset{•}{p}\;{}^{Al}, \end{array}$$

(182) $$\begin{array}{ccc} \left(\lambda^{A}{}_{;k}u_{l}^{A}+\lambda^{A}u_{l;k}^{A}\right)\frac{1}{c^{2}}q^{Ak}u^{Al} & = & \lambda^{A}{}_{;k}q^{Ak}, \end{array}$$

(183) $$\begin{array}{ccc} -\left(\lambda^{A}{}_{;k}u_{l}^{A}+\lambda^{A}u_{l;k}^{A}\right)p^{A}h^{Akl} & = & -\lambda^{A}p^{A}u^{Ak}{}_{;k}, \end{array}$$

(184) $$\begin{array}{ccc} \left(\lambda^{A}{}_{;k}u_{l}^{A}+\lambda^{A}u_{l;k}^{A}\right)\pi^{Akl} & = & \lambda^{A}u_{l;k}^{A}\pi^{Akl}. \end{array}$$

Taking Equations (181), (182) and (184) into account, we obtain from Equation (93)${}_{1}$

(185) $$\begin{array}{c} {\left(\lambda^{A}u_{l}^{A}\right)}_{;k}\left(T^{Akl}-\frac{1}{c^{2}}e^{A}u^{Ak}u^{Al}+p^{A}h^{Akl}\right)\;=\;\\ =\;-\lambda^{A}u_{l}^{A}\overset{•}{p}\;{}^{Al}+\lambda^{A}{}_{;k}q^{Ak}+\lambda^{A}u^{A}{}_{l;k}\pi^{Akl}.\; \end{array}$$

Replacing the second row of Equation (157) by the LHS of Equation (185) yields the entropy production of vanishing multi-temperature relaxation and vanishing spin by taking Equation (126) into account

(186) $$\begin{array}{ccc} \mathrm{without}\;\mathrm{spin}:\;\sigma_{0}^{A} & = & \left(\lambda^{A}\mu^{A}\right){}_{;k}J^{Am}h_{m}^{Ak}+\lambda^{A}\mu^{A}\left({}^{\left(in\right)}\Gamma^{A}-{\left(J^{Am}h_{m}^{Ak}\right)}_{;k}\right)+\\ & & +{\left(\lambda^{A}u_{l}^{A}\right)}_{;k}\left(T^{Akl}-\frac{1}{c^{2}}e^{A}u^{Ak}u^{Al}+p^{A}h^{Akl}\right)+\\ & & +\;{\left(\frac{1}{\Theta^{A}}u^{m}h_{ml}^{A}\right)}_{;k}\left(\frac{1}{c^{2}}e^{A}u^{Ak}u^{Al}+\frac{1}{c^{2}}q^{Ak}u^{Al}+t^{Akl}\right). \end{array}$$

It is evident that

(187) $${\left(\lambda^{A}u_{l}^{A}\right)}_{;k}\left(T^{Akl}-\frac{1}{c^{2}}e^{A}u^{Ak}u^{Al}+p^{A}h^{Akl}\right)\;=\;0$$

is not a sufficient condition for equilibrium because the equilibrium conditions in Equations (173)${}_{2}$, (161)${}_{1}$ and (164)${}_{1}$ are not necessarily satisfied and the entropy production in Equation (186) does not vanish. If the energy–momentum tensor is symmetric, Equation (187) results in

(188) $$T^{Akl}=T^{Alk}:\;\left[{\left(\lambda^{A}u_{l}^{A}\right)}_{;k}+{\left(\lambda^{A}u_{k}^{A}\right)}_{;l}\right]\left(T^{Akl}-\frac{1}{c^{2}}e^{A}u^{Ak}u^{Al}+p^{A}h^{Akl}\right)\;=\;0,$$

an expression which as well as Equation (187) is not sufficient for equilibrium. Consequently, the *Killing relation of the 4-temperature* $\lambda^{A}u_{l}^{A}$

(189) $$\left[{\left(\lambda^{A}u_{l}^{A}\right)}_{;k}+{\left(\lambda^{A}u_{k}^{A}\right)}_{;l}\right]\;=\;0$$

is also not sufficient for equilibrium (a fact which is well-known [12]).

If equilibrium is presupposed, the equilibrium conditions in Equations (173)${}_{2}$, (161)${}_{1}$ and (164)${}_{1}$ are satisfied, the entropy production vanishes and

(190) $$\begin{array}{c} \mathrm{without}\mathrm{spin}:\;{\left(\lambda^{A}u_{l}^{A}\right)}_{;k}^{eq}{\left(T^{Akl}-\frac{1}{c^{2}}e^{A}u^{Ak}u^{Al}+p^{A}h^{Akl}\right)}^{eq}\;=\;0, \end{array}$$

(191) $$\begin{array}{c} T_{eq}^{Akl}=T_{eq}^{Alk}:\;{\left[{\left(\lambda^{A}u_{l}^{A}\right)}_{;k}+{\left(\lambda^{A}u_{k}^{A}\right)}_{;l}\right]}^{eq}{\left(T^{Akl}-\frac{1}{c^{2}}e^{A}u^{Ak}u^{Al}+p^{A}h^{Akl}\right)}^{eq}\;=\;0 \end{array}$$

are necessary conditions (but, as discussed, not sufficient conditions) for equilibrium according to Equation (186), if the spin is ignored. If the spin is taken into account, Equation (190) results by use of the fifth row of Equation (157) in

(192) $$\begin{array}{c} {\left(\lambda^{A}u_{l}^{A}\right)}_{;k}^{eq}{\left(T^{Akl}-\frac{1}{c^{2}}e^{A}u^{Ak}u^{Al}+p^{A}h^{Akl}\right)}^{eq}\;=\;\\ =\;-{\left(\frac{\overset{•}{u}\;{}_{b}^{A}}{\Theta^{A}}\right)}_{;k}^{eq}\Xi_{eq}^{Akb}+\frac{\overset{•}{u}\;{}_{b}^{Aeq}}{\Theta_{eq}}u_{a}^{Aeq}{\left(s^{Akab}{}_{;k}\right)}^{eq}.\; \end{array}$$

There are different possibilities to satisfy Equations (190) and (191), which are discussed in the next section.

#### 6.6.3. The Gradient of the 4-Temperature

The necessary condition for equilibrium ignoring spin in Equation (190) can be differently satisfied generating different types of equilibria. There are three possibilities:

If equilibrium exists, one of the following three conditions is valid:

(193) $$\begin{array}{ccc} {\left(\lambda^{A}u_{l}^{A}\right)}_{;k}^{eq} & = & 0\;⟶\;\lambda_{;k}^{Aeq}u_{l}^{Aeq}+\lambda_{eq}^{A}u_{l;k}^{Aeq}\;=\;0, \end{array}$$

(194) $$\begin{array}{ccc} T_{eq}^{Akl} & = & \frac{1}{c^{2}}e_{eq}^{A}u_{eq}^{Ak}u_{eq}^{Al}-p_{eq}^{A}h_{eq}^{Akl}, \end{array}$$

(195) $$\begin{array}{ccc} {\left(\lambda^{A}u_{l}^{A}\right)}_{;k}^{eq}\;\neq \;0 & \wedge & \left[T_{eq}^{Akl}\;\neq \;\frac{1}{c^{2}}e_{eq}^{A}u_{eq}^{Ak}u_{eq}^{Al}-p_{eq}^{A}h_{eq}^{Akl}\right],\;\mathrm{and}\;\mathrm{Equation}\;(\mathrm{l90})\;\mathrm{is}\;\mathrm{valid}. \end{array}$$

Multiplication of Equation (193)${}_{2}$ with $u_{eq}^{Al}$ results in

(196) $$\lambda_{;k}^{Aeq}\;=\;0\;\wedge \;u_{l;k}^{Aeq}\;=\;0,$$

which means Equation (193) represents an intensified equilibrium because, in addition to the usual equilibrium conditions mentioned in Section 6.6.1, Equation (196) is valid.

If Equation (194) is valid, the equilibrium exists in a perfect material whose entropy production is zero. If the considered material is not perfect and if the equilibrium is not intensified, Equation (195) is valid, and the question arises on whether Equation (190) can be valid under these constraints. To answer this question, we consider Equations (180)–(184) in equilibrium. According to the equilibrium conditions, we obtain

(197) $$\begin{array}{ccc} {\left(\lambda_{;k}^{A}u_{l}^{A}+\lambda^{A}u_{l;k}^{A}\right)}^{eq}\frac{1}{c^{2}}e^{A}u_{eq}^{Ak}u_{eq}^{Al}\; & = & 0, \end{array}$$

(198) $$\begin{array}{ccc} {\left(\lambda_{;k}^{A}u_{l}^{A}+\lambda^{A}u_{l;k}^{A}\right)}^{eq}u_{eq}^{Ak}p_{eq}^{Al}\; & = & 0, \end{array}$$

(199) $$\begin{array}{ccc} {\left(\lambda_{;k}^{A}u_{l}^{A}+\lambda^{A}u_{l;k}^{A}\right)}^{eq}\frac{1}{c^{2}}q_{eq}^{Ak}u_{eq}^{Al}\; & = & 0, \end{array}$$

(200) $$\begin{array}{ccc} -{\left(\lambda_{;k}^{A}u_{l}^{A}+\lambda^{A}u_{l;k}^{A}\right)}^{eq}p^{A}h_{eq}^{Akl}\; & = & 0, \end{array}$$

(201) $$\begin{array}{ccc} {\left(\lambda_{;k}^{A}u_{l}^{A}+\lambda^{A}u_{l;k}^{A}\right)}^{eq}\pi_{eq}^{Akl}\; & = & 0. \end{array}$$

Summing up Equations (197)–(201) yields

(202) $$\left(\lambda^{A}u_{l}^{A}\right){}_{;k}^{eq}T_{eq}^{Akl}\;=\;0.$$

Consequently, Equation (190) is satisfied because each of the three terms vanishes for its own, thus being compatible with Equation (195). If an ${}^{A}$-component of a mixture is in equilibrium, two types of equilibria can occur: one in an arbitrary material showing the usual equilibrium conditions and another one which shows beyond the the usual equilibrium conditions vanishing temperature gradient and vanishing 4-velocity gradient according to Equation (196).

It is evident that a 1-component system which does not interact with other components is a special case included in the theory of an ${}^{A}$-component in the mixture. This case is discussed in the next section.

## 7. Special Case: 1-Component System

### 7.1. Entropy Flux, -Supply and -Density

A 1-component system (that is not a mixture which is a multi-component system by definition) can be described by setting equal all component indices of a multi-component system

(203) A,B,C,…,Z⟶0,

and, for brevity, we omit this common index 0. Then, the basic fields of a 1-component system are according to Equation (2)

(204) $$\mathrm{rest}\;\mathrm{mass}\;\mathrm{density}\;\mathrm{and}\;4-\mathrm{velocity}:\;\{\varrho ,u_{k}\}.$$

The equations (Equation (5)) of Setting I change into identities. According to Equations (9)${}_{1}$, (15)${}_{1}$, (17)${}_{2}$, (25) and (84)${}_{1}$, we have

(205) $$f\;=\;1,\;J_{k}\;=\;0,\;w\;=\;0,\;T^{kl}{}_{;k}\;=\;k^{l}\;S^{kab}{}_{;k}\;=\;\frac{1}{c^{2}}m^{ab}.$$

The Lagrange multipliers become according to Equations (126), (130), (132) and (137)

(206) $$\lambda \;=\;\frac{\nu }{\Theta },\;\kappa \;=\;\frac{\mu }{\Theta },\;\lambda^{k}\;=\;0,\;\Lambda_{a}\;=\;\frac{{\overset{•}{u}}_{a}}{\Theta }\;\Lambda_{ab}\;=\;0.$$

The entropy density in Equation (138) and the state space in Equation (113) are as in equilibrium of the ${}^{A}$-component in Equation (176)

(207) $$s\;=\;\frac{1}{\Theta }\left(\mu \varrho +\nu \left(e+p\right)+{\overset{•}{u}}_{m}\frac{1}{c^{2}}\Xi^{m}\right),\;z\;=\;\left(\varrho ,e,\Xi^{m}\right).$$

The entropy flux in Equation (139), the entropy supply in Equation (140) and the entropy production in Equation (157) are by taking Equation (185) into account (there are no chemical reactions in 1-component systems)

(208) $$\begin{array}{c} s^{k}\;=\;\frac{1}{\Theta }\left(\nu q^{k}+{\overset{•}{u}}_{m}\Xi^{km}\right),\;\varphi \;=\;\frac{1}{\Theta }\left(\mu \;{}^{\left(ex\right)}\Gamma +\nu u_{l}k^{l}+{\overset{•}{u}}_{b}u_{a}\frac{1}{c}m^{ab}\right), \end{array}$$

(209) $$\begin{array}{c} \sigma \;=\;{\left(\frac{\nu }{\Theta }u_{l}\right)}_{;k}\left(T^{kl}-\frac{1}{c^{2}}eu^{k}u^{l}+ph^{kl}\right)+{\left(\frac{\overset{•}{u}\;{}_{b}}{\Theta }\right)}_{;k}\Xi^{kb}-\frac{\overset{•}{u}\;{}_{b}}{\Theta }u_{a}s^{kab}{}_{;k}. \end{array}$$

According to Section 3.4, the (3+1)-components of the mixture change into the corresponding quantities of the 1-component system. The necessary equilibrium conditions of a 1-component system are equal to those of an ${}^{A}$-component in the mixture (in equilibrium: “all cats are grey”).

### 7.2. Equilibrium and Reversibility

Vanishing entropy production out of equilibrium

(210) $$\sigma^{rev}\;=\;0,\;\left(s^{k}\;\neq \;0\;\vee \;\varphi \;\neq \;0\right)$$

belongs to reversible processes and vice versa [29]. According to Equation (209),

(211) $$\begin{array}{c} {\left(\frac{\nu }{\Theta }u_{l}\right)}_{;k}^{rev}{\left(T^{kl}-\frac{1}{c^{2}}eu^{k}u^{l}+ph^{kl}\right)}^{rev}\;=\;\\ =\;-{\left(\frac{\overset{•}{u}\;{}_{b}}{\Theta }\right)}_{;k}^{rev}\Xi_{rev}^{kb}+{\left(\frac{\overset{•}{u}\;{}_{b}}{\Theta }u_{a}\right)}^{rev}{\left(s^{kab}{}_{;k}\right)}^{rev}.\; \end{array}$$

is sufficient and necessary for vanishing entropy production in 1-component systems. However, concerning equilibrium, Equation (211) is, as well as Equation (192), only necessary but not sufficient for it. Thus, all results of Section 6.6.3 change into those of a 1-component system, if the component index ${}^{A}$ is omitted, *eq* is replaced by *rev*, equilibrium is not presupposed and the generated expressions belong to reversible processes and vice versa.

Comparing Equation (211) with Equation (192) and ignoring the spin, the derivative of the 4-temperature and the Killing relation of the 4-temperature

(212) $$\mathrm{without}\mathrm{spin}:\;{\left(\lambda u_{l}\right)}_{;k}^{rev}\;=\;0,\;\mathrm{or}\;T^{kl}=T^{lk}\;:\;{\left({\left(\lambda u_{l}\right)}_{;k}+{\left(\lambda u_{k}\right)}_{;l}\right)}^{rev}\;=\;0$$

are rather conditions for reversible processes in 1-component systems because the entropy production is enforced to be zero without existing equilibrium. Independently of the 4-temperature, we obtain the well-known fact [30] that, according to Equation (209), all processes of perfect materials are reversible in 1-component systems without spin

(213) $$T_{per}^{kl}\;:=\;\frac{1}{c^{2}}eu^{k}u^{l}-ph^{kl}\;⟶\;\sigma_{per}\;=\;{\left(\frac{\overset{•}{u}\;{}_{b}}{\Theta }\right)}_{;k}\Xi^{kb}-\frac{\overset{•}{u}\;{}_{b}}{\Theta }u_{a}s^{kab}{}_{;k}.$$

## 8. Thermodynamics of a Mixture

According to the mixture axiom in Section 2.2, the balance equations of a mixture look like those of a 1-component system. However, a mixture as a whole behaves differently from the interacting ${}^{A}$-component in the mixture and also differently from a 1-component system, both of which are discussed in Section 6 and Section 7. Because the interaction between the components is still existing in the mixture, the diffusion fluxes and also the multi-temperature relaxation do not vanish as in 1-component systems. Because component indices ${}^{A}$ do not appear in the description of mixtures, they are summed up in contrast to 1-component systems for which they vanish. Settings I–III enforce the mixture axiom resulting in

(214) $$\begin{array}{ccc} \mathrm{mass}\;\mathrm{balance}: & \; & N^{Ak}{}_{;k}\;=\;\Gamma^{A}\;⟶\;N^{k}{}_{;k}\;=\;\Gamma , \end{array}$$

(215) $$\begin{array}{ccc} \mathrm{energy}\;\mathrm{balance}: & \; & u_{l}^{A}T^{Akl}{}_{;k}ß=\;\Omega^{A}\;⟶\;u_{l}T^{kl}{}_{;k}\;=\;\Omega , \end{array}$$

(216) $$\begin{array}{ccc} \mathrm{momentum}\;\mathrm{balance}: & \; & h_{l}^{Am}T^{Akl}{}_{;k}\;=\;\Omega^{Am}\;⟶\;h_{l}^{m}T^{kl}{}_{;k}\;=\;\Omega^{m}, \end{array}$$

(217) $$\begin{array}{ccc} \mathrm{spin}\;\mathrm{balance}: & \; & S^{Akab}{}_{;k}\;=\;\frac{1}{c^{2}}m^{Aab}\;⟶\;S^{kab}{}_{;k}\;=\;\frac{1}{c^{2}}m^{ab.} \end{array}$$

Settings I–XI are concerned with the balance equations (Equations (214)–(217)), with the entropy density, the entropy flux density, the entropy supply, the Lagrange multipliers and the multi-temperature relaxation. Obviously, we need an additional setting concerning the entropy of the mixture, which is formulated in the next section.

### 8.1. Additivity of 4-Entropies

#### 8.1.1. Entropy Density and -Flux

Similar to additivity of the mass flux densities in Equation (5)${}_{1}$, the energy–momentum tensors in Equation (42)${}_{2}$ and the spin tensors in Equation (78) of the ${}^{A}$-components, we demand that also the 4-entropies are additive

(218) $$\begin{array}{c} \mathrm{Setting}\mathrm{XII}:\;\\ S^{k}\;\overset{•}{=}\;\sum_{A}S^{Ak}.\; \end{array}$$

Consequently, we obtain from Equation (159) by use of Equations (22)${}_{2}$ and (41)

(219) $$\begin{array}{c} S^{k}=\sum_{A}\{\frac{\mu^{A}}{\Theta^{A}}\left(\left(1-w^{A}\right)N^{Ak}+J^{Ak}\right)+\frac{1}{\Theta^{A}}\left(\nu^{A}Q^{Ak}+u_{m}\tau^{Akm}\right)+\frac{\nu^{A}}{\Theta^{A}}p^{A}u^{Ak}+\;\\ \;\;+\frac{1}{\Theta^{A}}\overset{•}{u}\;{}_{m}^{A}\left(\frac{1}{c^{2}}\Xi^{Am}u^{Ak}+\Xi^{Akm}\right)+\frac{1}{\Theta^{A}}\overset{•}{u}\;{}_{[a}^{A}u_{b]}\left(\frac{1}{c^{2}}s^{Aab}u^{Ak}+s^{Akab}\right)+G^{A}u^{Ak}\}.\; \end{array}$$

According to Equation (87), we obtain the entropy density and the entropy flux density of the mixture by use of Equations (16), (9)${}_{1}$, (20) and (45)${}_{1}$

(220) $$\begin{array}{ccc} S^{k}u_{k}=c^{2}s & = & \sum_{A}\{\frac{\mu^{A}}{\Theta^{A}}\left(1-w^{A}\right)\varrho^{A}c^{2}f^{A}+\frac{1}{\Theta^{A}}\left(\nu^{A}Q^{Ak}+u_{p}\tau^{Akp}\right)u_{k}+,\\ & & \;+\left(\frac{\nu^{A}}{\Theta^{A}}p^{A}+G^{A}\right)c^{2}f^{A}+\frac{1}{\Theta^{A}}\overset{•}{u}\;{}_{m}^{A}\left(\Xi^{Am}f^{A}+\Xi^{Akm}u_{k}\right)+\\ & & \;+\frac{1}{\Theta^{A}}\overset{•}{u}\;{}_{[a}^{A}u_{b]}\left(s^{Aab}f^{A}+s^{Akab}u_{k}\right)\}, \end{array}$$

(221) $$\begin{array}{ccc} S^{k}h_{k}^{m}=s^{m} & = & \sum_{A}\{\frac{\mu^{A}}{\Theta^{A}}J^{Am}+\frac{1}{\Theta^{A}}\left(\nu^{A}Q^{Ak}+u_{p}\tau^{Akp}\right)h_{k}^{m}\\ & & \;+\left(\frac{\nu^{A}}{\Theta^{A}}p^{A}+G^{A}\right)c^{2}g^{Am}+\frac{1}{\Theta^{A}}\overset{•}{u}\;{}_{p}^{A}\left(\Xi^{Ap}g^{Am}+\Xi^{Akp}h_{k}^{m}\right)+\\ & & \;+\frac{1}{\Theta^{A}}\overset{•}{u}\;{}_{[a}^{A}u_{b]}\left(\frac{1}{c^{2}}s^{Aab}g^{Am}+s^{Akab}h_{k}^{m}\right)\}. \end{array}$$

Taking Equation (43) into consideration, we introduce by comparing with Equations (220) and (221)

(222) $$\begin{array}{c} \mathrm{Setting}\mathrm{XIII}:\;\\ \nu^{A}\;\overset{•}{=}\;f^{A}.\; \end{array}$$

With this setting, the expressions of the entropy density and the entropy flux of the mixture correspond to those which are generated by the additivity of the energy–momentum tensors: Equations (48)–(55).

Finally, we obtain the entropy and entropy flux densities of the mixture

(223) $$\begin{array}{ccc} s & = & \sum_{A}\{\frac{\mu^{A}}{\Theta^{A}}\left(1-w^{A}\right)\varrho^{A}f^{A}+\frac{1}{c^{2}}\frac{1}{\Theta^{A}}\left(f^{A}Q^{Ak}+u_{p}\tau^{Akp}\right)u_{k}+\\ & & \;+\left(\frac{f^{A}}{\Theta^{A}}p^{A}+G^{A}\right)f^{A}+\frac{1}{\Theta^{A}}\overset{•}{u}\;{}_{m}^{A}\frac{1}{c^{2}}\left(\Xi^{Am}f^{A}+\Xi^{Akm}u_{k}\right)+\\ & & \;+\frac{1}{\Theta^{A}}\overset{•}{u}\;{}_{[a}^{A}u_{b]}\frac{1}{c^{2}}\left(s^{Aab}f^{A}+s^{Akab}u_{k}\right)\}, \end{array}$$

(224) $$\begin{array}{ccc} s^{m} & = & \sum_{A}\{\frac{\mu^{A}}{\Theta^{A}}J^{Am}+\frac{1}{\Theta^{A}}\left(f^{A}Q^{Ak}+u_{p}\tau^{Akp}\right)h_{k}^{m}\\ & & \;+\left(\frac{f^{A}}{\Theta^{A}}p^{A}+G^{A}\right)c^{2}g^{Am}+\frac{1}{\Theta^{A}}\overset{•}{u}\;{}_{p}^{A}\left(\Xi^{Ap}g^{Am}+\Xi^{Akp}h_{k}^{m}\right)+\\ & & \;+\frac{1}{\Theta^{A}}\overset{•}{u}\;{}_{[a}^{A}u_{b]}\left(\frac{1}{c^{2}}s^{Aab}g^{Am}+s^{Akab}h_{k}^{m}\right)\}. \end{array}$$

These expressions of the entropy and entropy flux densities of the mixture are direct results of Setting XII in Equation (218). They are considered in Section 8.2.

#### 8.1.2. Entropy Supply and Production Density

From Equations (218) and (86)${}_{2}$ follows the entropy balance equation of the mixture

(225) $$S^{k}{}_{;k}\;=\;\sum_{A}S^{Ak}{}_{;k}\;=\;\sum_{A}\left(\sigma^{A}+\varphi^{A}\right)\;={\;}^{⋄}\;\sigma {+}^{⋄}\;\;\varphi ,$$

satisfying the mixture axiom. Accepting the additivity of the entropy supplies of the ${}^{A}$-components (supplies are caused by external influences, productions by internal ones, which is why they do not mix up)

(226) $$\begin{array}{c} \mathrm{Setting}\mathrm{XIV}:\;\\ {}^{⋄}\;\;\varphi \;\overset{•}{=}\;\sum_{A}\varphi^{A},\; \end{array}$$

we obtain from Equation (225) the additivity of the entropy productions of the ${}^{A}$-components

(227) $${}^{⋄}\;\sigma \;=\;\sum_{A}\sigma^{A}.\;$$

The entropy supply of the mixture follows from Equations (156), (226) and (222)

(228) $$\begin{array}{c} {}^{⋄}\;\;\varphi \;=\;\sum_{A}\{\frac{1}{\Theta^{A}}[\mu^{A}{}^{\left(ex\right)}\Gamma^{A}+\left(f^{A}u_{l}^{A}+u_{m}h_{l}^{Am}\right)k^{Al}+\;\\ +\left(\overset{•}{u}{}_{b}^{A}u_{a}^{A}+\overset{•}{u}\;{}_{[m}^{A}u_{n]}h_{a}^{Am}h_{b}^{An}\right)\frac{1}{c^{2}}m^{Aab}]+u^{Ak}{}_{;k}G^{A}\}. \end{array}$$

The entropy production of the mixture follows from Equations (157), (227) and (222)

(229) $$\begin{array}{ccc} {}^{⋄}\;\sigma \; & = & \sum_{A}\{{\left(\frac{\mu^{A}}{\Theta^{A}}\right)}_{;k}J^{Am}h_{m}^{Ak}+\frac{\mu^{A}}{\Theta^{A}}\left[{}^{\left(in\right)}\;\Gamma^{A}+\left(J^{Am}h_{m}^{Ak}\right){}_{;k}\right]+\\ & & +{\left(\frac{f^{A}}{\Theta^{A}}\right)}_{;k}q^{Ak}+\frac{f^{A}}{\Theta^{A}}u^{A}{}_{l;k}\left(\pi^{Akl}+u^{Ak}p^{Al}\right)+\\ & & +\;{\left(\frac{1}{\Theta^{A}}u^{m}h_{ml}^{A}\right)}_{;k}\left(\frac{1}{c^{2}}e^{A}u^{Ak}u^{Al}+\frac{1}{c^{2}}q^{Ak}u^{Al}+t^{Akl}\right)+\\ & & +\sum_{B}H^{AB}{\left(\frac{1}{\Theta^{A}}-\frac{1}{\Theta^{B}}\right)}^{{}^{•}}+\\ & & +{\left(\frac{\overset{•}{u}\;{}_{b}^{A}}{\Theta^{A}}\right)}_{;k}\Xi^{Akb}-\frac{\overset{•}{u}\;{}_{b}^{A}}{\Theta^{A}}u_{a}^{A}s^{Akab}{}_{;k}+\\ & & +\left(\frac{1}{\Theta^{A}}\overset{•}{u}\;{}_{[m}^{A}u_{n]}h_{a}^{Am}h_{b}^{An}\right){}^{•}\frac{1}{c^{4}}u^{A[a}\Xi^{Ab]}+\\ & & +{\left(\frac{1}{\Theta^{A}}\overset{•}{u}\;{}_{[m}^{A}u_{n]}h_{a}^{Am}h_{b}^{An}\right)}_{;k}\left(\frac{1}{c^{2}}u^{A[a}\Xi^{Akb]}+s^{Akab}\right)\}. \end{array}$$

### 8.2. Partial and Mixture Temperatures

We now consider the positive term in the second row of the entropy density in Equation (223)

(230) $$\sum_{A}\frac{1}{\Theta^{A}}{\left(f^{A}\right)}^{2}p^{A}\;=\;\frac{1}{{}^{⋄}\;\;\Theta }\sum_{A}{\left(f^{A}\right)}^{2}p^{A}\;>\;0$$

by which a mixture temperature ${}^{⋄}\;\;\Theta$ can be defined. This mixture temperature is only a formal quantity because it is not evident that a thermometer exists which measures ${}^{⋄}\;\;\Theta$: the partial temperatures are internal contact variables [31] measured by thermometers, which are selective for the temperature $\Theta^{A}$ of the corresponding ${}^{A}$-component. It is evident that the measured mixture temperature is a certain mean value of the partial temperatures of the components of the mixture [32,33,34], but this measured mean value may depend on the individual thermometer and may be different from ${}^{⋄}\;\;\Theta$, which means the measured temperature is not unequivocal. Different definitions of the mixture temperature can be found in literature [35]. However, a unique mixture temperature—independent of thermometer selectivities or arbitrary definitions—is given in the case of *multi-temperature relaxation equilibrium* in Equation (175). This case is often silently presupposed in the literature, if only one temperature is used in multi-component non-equilibrium systems. Only this sure case is considered in the sequel.

We now introduce the mixture quantities e and $q^{m}$ to the entropy density s and the entropy flux density $s^{m}$. According to Equations (48) and (50), we obtain

(231) $$\frac{1}{c^{2}}\sum_{A}\frac{1}{{}^{⋄}\;\;\Theta }\left(Q^{Ak}f^{A}+u_{p}\tau^{Akp}\right)u_{k}\;=\;\frac{e}{{}^{⋄}\;\;\Theta },\;\sum_{A}\frac{1}{{}^{⋄}\;\;\Theta }\left(Q^{Ak}f^{A}+u_{p}\tau^{Akp}\right)h_{k}^{m}\;=\;\frac{q^{m}}{{}^{⋄}\;\;\Theta }.$$

Taking Equations (230) and (231) into account, Equations (223) and (224) result in

(232) $$\begin{array}{ccc} s & = & \frac{e}{{}^{⋄}\;\;\Theta }+\sum_{A}\{\frac{\mu^{A}}{\Theta^{A}}\left(1-w^{A}\right)\varrho^{A}f^{A}+\frac{1}{c^{2}}\left(\frac{1}{\Theta^{A}}-\frac{1}{{}^{⋄}\;\;\Theta }\right)\left(f^{A}Q^{Ak}+u_{p}\tau^{Akp}\right)u_{k}+\\ & & \;+\left(\frac{f^{A}}{{}^{⋄}\;\;\Theta }p^{A}+G^{A}\right)f^{A}+\frac{1}{\Theta^{A}}\overset{•}{u}\;{}_{m}^{A}\frac{1}{c^{2}}\left(\Xi^{Am}f^{A}+\Xi^{Akm}u_{k}\right)+\\ & & \;+\frac{1}{\Theta^{A}}\overset{•}{u}\;{}_{[a}^{A}u_{b]}\frac{1}{c^{2}}\left(s^{Aab}f^{A}+s^{Akab}u_{k}\right)\}, \end{array}$$

(233) $$\begin{array}{ccc} s^{m} & = & \frac{q^{m}}{{}^{⋄}\;\;\Theta }+\sum_{A}\{\frac{\mu^{A}}{\Theta^{A}}J^{Am}+\left(\frac{1}{\Theta^{A}}-\frac{1}{{}^{⋄}\;\;\Theta }\right)\left(f^{A}Q^{Ak}+u_{p}\tau^{Akp}\right)h_{k}^{m}+\\ & & \;+\left(\frac{f^{A}}{\Theta^{A}}p^{A}+G^{A}\right)c^{2}g^{Am}+\frac{1}{\Theta^{A}}\overset{•}{u}\;{}_{p}^{A}\left(\Xi^{Ap}g^{Am}+\Xi^{Akp}h_{k}^{m}\right)+\\ & & \;+\frac{1}{\Theta^{A}}\overset{•}{u}\;{}_{[a}^{A}u_{b]}\left(\frac{1}{c^{2}}s^{Aab}g^{Am}+s^{Akab}h_{k}^{m}\right)\}. \end{array}$$

It is evident that partial temperatures of the components appear in all four quantities referring to mixture entropy: entropy density in Equation (232), entropy flux density in Equation (233), entropy supply in Equation (228) and entropy production density in Equation (229). These expressions are of a more simple shape, if the mixture is in a multi-temperature equilibrium which is considered in the next section.

### 8.3. Multi-Temperature Relaxation Equilibrium

#### 8.3.1. Entropy and Entropy Flux Densities

Presupposing multi-temperature relaxation equilibrium in Equation (175), the entropy density in Equation (232) and the entropy flux density in Equation (233) become

(234) $$\begin{array}{ccc} s & = & \frac{e}{{}^{⋄}\;\;\Theta }+\frac{1}{{}^{⋄}\;\;\Theta }\sum_{A}\{\mu^{A}\left(1-w^{A}\right)\varrho^{A}f^{A}+{\left(f^{A}\right)}^{2}p^{A}+\\ & & \;\;+\overset{•}{u}{}_{m}^{A}\frac{1}{c^{2}}\left(\Xi^{Am}f^{A}+\Xi^{Akm}u_{k}\right)+\overset{•}{u}\;{}_{[a}^{A}u_{b]}\frac{1}{c^{2}}\left(s^{Aab}f^{A}+s^{Akab}u_{k}\right)\}, \end{array}$$

(235) $$\begin{array}{ccc} s^{m} & = & \frac{q^{m}}{{}^{⋄}\;\;\Theta }+\frac{1}{{}^{⋄}\;\;\Theta }\sum_{A}\{\mu^{A}J^{Am}+f^{A}p^{A}c^{2}g^{Am}+\\ & & \;\;+\overset{•}{u}{}_{p}^{A}\left(\Xi^{Ap}g^{Am}+\Xi^{Akp}h_{k}^{m}\right)+\overset{•}{u}\;{}_{[a}^{A}u_{b]}\left(\frac{1}{c^{2}}s^{Aab}g^{Am}+s^{Akab}h_{k}^{m}\right)\}.\; \end{array}$$

The first term in the sum of Equation (234) can be exploited by use of the mean value theorem according to Equations (18) and (9)${}_{1,2}$

(236) $$\sum_{A}\mu^{A}\left(1-w^{A}\right)\varrho^{A}f^{A}\;={\;}^{⋄}\;\mu \sum_{A}\left(1-w^{A}\right)f^{A}\varrho^{A}\;={\;}^{⋄}\;\mu \sum_{A}{\left(f^{A}\right)}^{3}\varrho^{A}$$

Consequently, the chemical potential of the mixture is

(237) $${}^{⋄}\;\;\mu \;:=\;\sum_{A}\mu^{A}\frac{\left(1-w^{A}\right)\varrho^{A}f^{A}}{\sum_{B}\left(1-w^{B}\right)\varrho^{B}f^{B}},$$

and the entropy density of the mixture in Equation (234) yields

(238) $$\begin{array}{ccc} s & = & \frac{1}{{}^{⋄}\;\;\Theta }e+\frac{1}{{}^{⋄}\;\;\Theta }{}^{⋄}\;\mu \sum_{A}{\left(f^{A}\right)}^{3}\varrho^{A}+\frac{1}{{}^{⋄}\;\;\Theta }\sum_{A}p^{A}{\left(f^{A}\right)}^{2}+\\ & & \;+\sum_{A}\left\{\overset{•}{u}\;{}_{m}^{A}\frac{1}{c^{2}}\left(\Xi^{Am}f^{A}+\Xi^{Akm}u_{k}\right)+\overset{•}{u}\;{}_{[a}^{A}u_{b]}\frac{1}{c^{2}}\left(s^{Aab}f^{A}+s^{Akab}u_{k}\right)\right\}. \end{array}$$

The entropy density in Equation (238) of the mixture in multi-temperature equilibrium is similarly constructed, but different from the expression in Equation (207) of a 1-component system: there are the energy, mass, pressure and spin terms.

#### 8.3.2. Entropy Production and -Supply

The entropy supply in Equation (228) results in

(239) $$\begin{array}{c} {}^{⋄}\;\;\varphi \;=\;\frac{1}{{}^{⋄}\;\;\Theta }\sum_{A}\{\mu^{A}{}^{\left(ex\right)}\Gamma^{A}+\left(f^{A}u_{l}^{A}+u_{m}h_{l}^{Am}\right)k^{Al}+\;\\ +\left(\overset{•}{u}{}_{b}^{A}u_{a}^{A}+\overset{•}{u}\;{}_{[m}^{A}u_{n]}h_{a}^{Am}h_{b}^{An}\right)\frac{1}{c^{2}}m^{Aab}\}. \end{array}$$

The entropy production density in Equation (229) becomes

(240) $$\begin{array}{ccc} {}^{⋄}\;\;\sigma \; & = & \sum_{A}\{{\left(\frac{\mu^{A}}{{}^{⋄}\;\;\Theta }\right)}_{;k}J^{Am}h_{m}^{Ak}+\frac{\mu^{A}}{{}^{⋄}\;\;\Theta }\left[{}^{\left(in\right)}\;\Gamma^{A}+\left(J^{Am}h_{m}^{Ak}\right){}_{;k}\right]+\\ & & +{\left(\frac{f^{A}}{{}^{⋄}\;\;\Theta }\right)}_{;k}q^{Ak}+\frac{f^{A}}{{}^{⋄}\;\;\Theta }u^{A}{}_{l;k}\left(\pi^{Akl}+u^{Ak}p^{Al}\right)+\\ & & +\;{\left(\frac{1}{{}^{⋄}\;\;\Theta }u^{m}h_{ml}^{A}\right)}_{;k}\left(\frac{1}{c^{2}}e^{A}u^{Ak}u^{Al}+\frac{1}{c^{2}}q^{Ak}u^{Al}+t^{Akl}\right)+\\ & & +{\left(\frac{\overset{•}{u}\;{}_{b}^{A}}{{}^{⋄}\;\;\Theta }\right)}_{;k}\Xi^{Akb}-\frac{\overset{•}{u}\;{}_{b}^{A}}{{}^{⋄}\;\;\Theta }u_{a}^{A}s^{Akab}{}_{;k}+\\ & & +\left(\frac{1}{{}^{⋄}\;\;\Theta }\overset{•}{u}\;{}_{[m}^{A}u_{n]}h_{a}^{Am}h_{b}^{An}\right){}^{•}\frac{1}{c^{4}}u^{A[a}\Xi^{Ab]}+\\ & & +{\left(\frac{1}{{}^{⋄}\;\;\Theta }\overset{•}{u}\;{}_{[m}^{A}u_{n]}h_{a}^{Am}h_{b}^{An}\right)}_{;k}\left(\frac{1}{c^{2}}u^{A[a}\Xi^{Akb]}+s^{Akab}\right)\}. \end{array}$$

The meaning of each individual term of Equation (240) is discussed above with regard to the ${}^{A}$-component according to Equation (157).

Entropy density in Equation (238), entropy flux density in Equation (235), entropy supply in Equation (239), entropy production density in Equation (240) and chemical potential in Equation (237) of the mixture are represented by sums of quantities of the ${}^{A}$-components. As expected, the (3+1)-components of the energy–momentum tensor cannot represent the mentioned thermodynamical quantities because diffusion fluxes, chemical potentials and temperature are not included in the energy–momentum tensor. From them, only the energy density e and the energy flux density $q^{m}$ of the mixture appear in entropy and entropy flux densities.

A temperature ${}^{⋄}\;\;\Theta$ of the mixture can be defined independently of multi-temperature equilibrium. According to Equation (230), $1{/}^{⋄}\;\;\Theta$ is a weighted mean value of the reciprocal partial temperatures of the components arranged with the partial pressures, a construction which seems very special. As mentioned in Section 8.2, a mixture temperature is not well defined because it depends on the component sensitivity of a thermometer.

### 8.4. Total Equilibrium

It is evident that the equilibrium conditions of a mixture follow from those of the ${}^{A}$-components which we considered in Section 6.6.1. Consequently, a demand of additional equilibrium conditions for mixtures is not necessary. Presupposing the equilibrium conditions of an ${}^{A}$-component (discussed in Section 6.6.1) and multi-temperature equilibrium, we start with the repetition

(241) $$\begin{array}{c} f_{eq}^{A}\;=\;1,\;w_{eq}^{A}\;=\;0,\;u_{k}^{Aeq}\;=\;u_{k}^{eq},\;g_{eq}^{Am}\;=\;0,{\;}^{\left(ex\right)}\Gamma_{eq}^{A}\;={\;}^{\left(in\right)}\Gamma_{eq}^{A}\;=\;0, \end{array}$$

(242) $$\begin{array}{c} e_{eq}\;=\;\sum_{A}e_{eq}^{A},\;q_{eq}^{m}\;=\;0,\;p_{eq}^{m}\;=\;\sum_{A}p_{eq}^{Am},\;T_{eq}^{jm}\;=\;\sum_{A}t_{eq}^{Ajm}. \end{array}$$

Taking Equations (241) and (242) into account, the entropy density in Equation (238) of the mixture in equilibrium and the entropy flux density in Equation (235) result in

(243) $$s_{eq}\;=\;\frac{1}{{}^{⋄}\;\;\Theta }e_{eq}+\frac{1}{{}^{⋄}\;\;\Theta }{}^{⋄}\;\mu \varrho +\frac{1}{{}^{⋄}\;\;\Theta }\sum_{A}p_{eq}^{A}+0,\;s_{eq}^{m}\;=\;0,$$

if the the shifting of the time derivative in Equations (172), (165), (72) and (73) is applied. Interestingly, the spin terms cancel in equilibrium.

The entropy supply in Equation (239) results in equilibrium

(244) $${}^{⋄}\;\;\varphi_{eq}\;=\;\frac{1}{{}^{⋄}\;\;\Theta }\sum_{A}\left\{u_{l}^{Aeq}k_{eq}^{Al}+\overset{•}{u}{}_{b}^{Aeq}u_{a}^{Aeq}\frac{1}{c^{2}}m_{eq}^{Aab}\right\}\;=\;0$$

according to Equation (178).

The entropy production density in Equation (240) results in equilibrium

(245) $${}^{⋄}\;\;\sigma_{eq}\;=\;\frac{1}{{}^{⋄}\;\;\Theta }\sum_{A}\left\{u^{Aeq}{}_{l;k}\pi_{eq}^{Akl}-\overset{•}{u}\;{}_{b}^{Aeq}u_{a}^{Aeq}{\left(s^{Akab}{}_{;k}\right)}^{eq}\right\}+\sum_{A}{\left(\frac{\overset{•}{u}\;{}_{b}^{A}}{{}^{⋄}\;\;\Theta }\right)}_{;k}\Xi^{Akb}\;=\;0,$$

according to Equation (179).

The vanishing entropy supply of the mixture in Equation (244) is satisfied by the equilibrium conditions in Equations (173)${}_{1}$, (178) and (170). The vanishing entropy production of the mixture in Equation (245) is satisfied by the equilibrium conditions in Equations (173)${}_{2}$, (161)${}_{1}$, (177), (179), (165) and (170).

From Equation (4) follows in equilibrium analogously to Equation (174)

(246) $$u_{eq}^{k}{}_{\;;k}\;=\;0.$$

### 8.5. (3+1)-Entropy-Components and Spin

If the spin is taken into consideration (that is absolutely necessary in General Relativity Theory, see Section 10), acceleration terms appear in the entropy density and production, Equations (238) and (240), and in the entropy flux density and supply, Equations (235) and (239). The four components in Equations (72) and (73) of the spin are differently distributed over the (3+1)-components of the entropy:

- The *entropy density* in Equation (154) of an ${}^{A}$-component depends on the spin density $s^{Aab}$ and on the spin density vector $\Xi^{Am}$, whereas the entropy density of the mixture in Equation (223) depends on the four spin quantities in Equations (72) and (73). In 1-component systems, the entropy density in Equation (207)${}_{1}$ depends only on the spin vector $\Xi^{m}$. In equilibrium, the entropy density is for all cases independent of the spin, Equations (176) and (243)${}_{1}$.
- The *entropy flux density* in Equation (139) of an ${}^{A}$-component depends on the couple stress $s^{Akab}$ and on the spin stress $\Xi^{Akm}$, whereas the entropy flux density of the mixture in Equation (235) depends on the four spin quantities in Equations (72) and (73). In 1-component systems, the entropy flux density in Equation (208)${}_{1}$ depends only on the spin vector $\Xi^{m}$. In equilibrium, the entropy flux density in Equations (176) and (243)${}_{2}$ vanishes and induces $q_{eq}^{Ak}=0$.
- The *entropy supply* of an ${}^{A}$-component in Equation (156) is as well independent of the spin as for the mixture in Equation (239) and for a 1-component system in Equation (208)${}_{2}$. The entropy supply vanishes in equilibrium, and a connection between the force density $k_{eq}^{Al}$ and the angular momentum density $m_{eq}^{Aab}$ is established, Equations (178) and (244).
- The *entropy production density* in Equations (157) and (240) does not depend on the spin density $s^{Aab}$ for an ${}^{A}$-component and for the mixture, but a dependence upon the three other (3+1)-spin-components exists. In 1-component systems, the entropy production density in Equation (209) depends on the spin stress $\Xi^{kb}$ and on the couple stress $s^{kab}$. The entropy production density vanishes in equilibrium, and a connection between the viscosity tensor $\pi_{eq}^{Akl}$ and the spin stress and the couple stress is established, Equations (179) and (245).

## 9. Balances, Constitutive Equations and the 2nd Law

Up to here, a special material was not taken into account: all considered relations are valid independently of the material which is described by constitutive equations supplementing the balance equations. Especially, the entropy productions in Equation (157) of the ${}^{A}$-component and Equation (240) of the mixture are not specified for particular materials. There are different possibilities for introducing constitutive equations (as an ansatz, or better by construction procedures [36,37]). Because constitutive equations are not in the center of our considerations, we restrict ourselves on the easiest ansatz, which only serves for elucidation of the problem: Balance equations are generally valid for all materials, which means they cannot be solved without choosing a special material characterized by constitutive equations which inserted into the balance equations transform these into a system of solvable differential equations for the wanted fields.

The entropy production of the ${}^{A}$-component in Equation (157) is a sum of two-piece products whose factors are so-called “fluxes” and “forces”. According to Equation (157), the ten fluxes are

(247) $$\begin{array}{c} Y^{A}\;=\;\{J^{Am}h_{m}^{Ak},\;\left[{}^{\left(in\right)}\Gamma^{A}-{\left(J^{Am}h_{m}^{Ak}\right)}_{;k}\right],\;q^{Ak},\;\left(\pi^{Akl}+u^{Ak}p^{Al}\right),\\ \left(\frac{1}{c^{2}}e^{A}u^{Ak}u^{Al}+\frac{1}{c^{2}}q^{Ak}u^{Al}+t^{Akl}\right),H^{AB},\Xi^{Akb},s^{Akab},\\ \frac{1}{c^{4}}u^{A[a}\Xi^{Ab]},\left(\frac{1}{c^{2}}u^{A[a}\Xi^{Akb]}+s^{Akab}\right)\}, \end{array}$$

and the corresponding ten forces are

(248) $$\begin{array}{c} X^{A}\;=\;\{\;{\left(\frac{\mu^{A}}{\Theta^{A}}\right)}_{;k},\frac{\mu^{A}}{\Theta^{A}},\;{\left(\frac{f^{A}}{\Theta^{A}}\right)}_{;k},\;\frac{f^{A}}{\Theta^{A}}u^{A}{}_{l,k},\;\\ {\left(\frac{1}{\Theta^{A}}u^{m}h_{ml}^{A}\right)}_{;k},\;{\left(\frac{1}{\Theta^{A}}-\frac{1}{\Theta^{B}}\right)}^{{}^{•}},\;{\left(\frac{\overset{•}{u}\;{}_{b}^{A}}{\Theta^{A}}\right)}_{;k},\;\frac{\overset{•}{u}\;{}_{b}^{A}}{\Theta^{A}}u_{a;k}^{A},\;\\ \left(\frac{1}{\Theta^{A}}\overset{•}{u}\;{}_{[m}^{A}u_{n]}h_{a}^{Am}h_{b}^{An}\right){}^{•},\;{\left(\frac{1}{\Theta^{A}}\overset{•}{u}\;{}_{[m}^{A}u_{n]}h_{a}^{Am}h_{b}^{An}\right)}_{;k}\}. \end{array}$$

The entropy production density in Equation (157) of an ${}^{A}$-component can be written as a scalar product of forces and fluxes

(249) $$\sigma^{A}\;=\;Y^{A}\cdot X^{A},$$

a relation which is valid independently of the material in consideration. The material is described by the dependence of the fluxes on the forces, by the constitutive equations

(250) $$Y^{A}\;=\;F^{A}\left(X^{A}\right)$$

which have to be introduced into the expression of the partial entropy production density in Equation (249) resulting in the entropy production density of the mixture by Equation (227)

(251) $$\sigma^{A}\;=\;F^{A}\left(X^{A}\right)\cdot X^{A}\;⟶{\;}^{⋄}\;\;\sigma \;=\;\sum_{A}F^{A}\left(X^{A}\right)\cdot X^{A}\;\geq \;0.$$

The inequality is caused by the Second Law, which states that the entropy production of the mixture is not negative after having inserted the constitutive equationsinto the general expression in Equation (229). Consequently, the Second Law represents a constraint for the constitutive equations (Equation (250)) [38], and it makes no sense to take the Second Law into consideration before the constitutive equations are inserted. The entropy production of sub-systems—here the ${}^{A}$-components in Equation (251)${}_{1}$— is not necessarily positive semi-definite. There are different methods for exploiting the dissipation inequality in Equation (251)${}_{2}$ [38,39], which are beyond this paper because special materials are here out of scope.

## 10. Special Case: General Relativity Theory

### 10.1. Extended Belinfante/Rosenfeld Procedure

The basic equations of General-Covariant Continuum Thermodynamics (GCCT) of a mixture (Section 8) (Equations (214)–(217) and (225))

(252) $$N^{k}{}_{;k}\;=\;\Gamma ,\;T^{kl}{}_{;k}\;=\;k^{l},\;S^{kab}{}_{;k}\;=\;\frac{1}{c^{2}}m^{ab},\;S^{k}{}_{;k}\;={\;}^{⋄\;}\sigma {+}^{⋄\;}\varphi$$

contain covariant derivatives depending on the geometry of the space-time in which the physical processes occur. Here, the pseudo-Riemannian space of General Relativity Theory (GRT) is chosen as a special case.

In GRT, as a consequence of Einstein’s equations

(253) $$R^{ab}-\frac{1}{2}g^{ab}R\;=\;\kappa \Theta^{ab}\;⟹\;\Theta^{ab}\;=\;\Theta^{ba},\;\Theta_{\;\;;b}^{ab}\;=\;0,$$

the gravitation generating energy–momentum tensor $\Theta^{ab}$ has to be symmetric and divergence-free ($R^{ab}$ is the Ricci tensor, $g^{ab}$ the metric, and $R=R^{m}{}_{m}$). According to Equations (42) and (28), the energy–momentum tensor of the mixture $T^{kl}$ may be neither symmetric nor divergence-free. The same is true for spin divergence $S^{kab}{}_{;k}$. Consequently, both tensors cannot serve as gravitation generating tensors in Einstein’s equations, and the question arises: How can the balance equations (Equations (252)${}_{2,3}$) be incorporated into the general-covariant framework of GRT? The answer to that question has been proved by the following extended Belinfante/Rosenfeld procedure whose special relativistic version is well known since a long time [40,41,42]. The general relativistic version is as follows: 

■ Proposition [43]: The general-covariant Belinfante/Rosenfeld procedure generates a symmetric and divergence-free tensor

(254) $${}^{†}\Theta^{ab}\;:=\;T^{ab}-\frac{1}{2}{\left[S^{kab}+S^{abk}+S^{bak}\right]}_{;k},$$

if the GCCT balances (B) in Equation (252)${}_{2,3}$ and the Mathisson–Papapetrou equations (MP) in Equations (255)${}_{2}$ and (256)${}_{2}$

(255) $$\begin{array}{ccc} \frac{1}{c^{2}}m^{ab} & \overset{{}_{B}}{=} & S^{kab}{}_{;k}\;\overset{{}_{MP}}{=}\;2T^{\left[ab\right]}, \end{array}$$

(256) $$\begin{array}{ccc} k^{b} & \overset{{}_{B}}{=} & T^{ab}{}_{;a}\;\overset{{}_{MP}}{=}\;\frac{1}{2}{\left[S^{kab}+S^{abk}+S^{bak}\right]}_{;k;a}\;=\;-\frac{1}{2}R_{klm}^{b}S^{klm} \end{array}$$

($R_{klm}^{b}$ is the curvature tensor) are valid as necessary constraints for the force density and the angular momentum. ■

The Mathisson–Papapetrou equations are general-covariant including the special-relativistic case, which is characterized by replacing the covariant derivatives by commuting partial ones and by $R_{klm}^{b}\equiv 0$.

Inserting Equation (255) into Equation (254) results in

(257) $${}^{†}\Theta^{ab}\;=\;T^{\left(ab\right)}-\frac{1}{2}{\left[S^{abc}+S^{bac}\right]}_{;c}\;=\;T^{\left(ab\right)}-S^{(ab)c}{}_{;c},$$

a tensor which is symmetric and divergence-free according to Equations (254) and (256)${}_{2}$.

The general-covariant Belinfante/Rosenfeld procedure transforms by use of the symmetric spin divergence $S^{(ab)c}{}_{;c}$ the not necessary divergence-free symmetric part of the energy–momentum tensor $T^{\left(ab\right)}$ into a symmetric and divergence-free tensor ${}^{†}\Theta^{ab}$. In other words, the energy–momentum tensor $T^{ab}$ (not necessary symmetric and divergence-free) is transformed into the mutant ${}^{†}\Theta^{ab}$ (symmetric and divergence-free) (this name was coined by H.-H. von Borzeszkowski).

The decisive step for connecting GRT and GCCT is the following usually used

(258) $$\begin{array}{c} \mathrm{Setting}\mathrm{XV}:\;\\ \Theta^{ab}\;\overset{•}{=}{\;}^{†}\Theta^{ab}.\; \end{array}$$

The mutant which is created by the Belinfante/Rosenfeld procedure is the gravitation generating energy–momentum tensor of Einstein’s equation (Equation (253)). According to Equation (252), the mixture (and not single components) determines the geometry.

### 10.2. Example: 2-Component Plain-Ghost Mixture

#### 10.2.1. The Plain Component

The balance equations defining the plain component (P) are according to Equations (1), (25) and (84)${}_{1}$

(259) $$\begin{array}{ccc} N_{k}^{P} & = & \varrho^{P}u_{k}^{P},\;N^{Pk}{}_{;k}\;\overset{{}_{B}}{=}\;0, \end{array}$$

(260) $$\begin{array}{ccc} T_{P}^{\left[ab\right]} & = & 0,\;T_{P}^{\left(ab\right)}{}_{;a}\;=\;0\;\overset{{}_{B}}{=}\;k_{P}^{b}, \end{array}$$

(261) $$\begin{array}{ccc} S_{P}^{abc} & = & 0,\;S_{P}^{kab}{}_{;k}\;=\;0\;\overset{{}_{B}}{=}\;\frac{1}{c^{2}}m_{P}^{ab}. \end{array}$$

By definition, the plain component is characterized by a symmetric and divergence-free energy–momentum tensor and vanishing spin. According to Equations (255) and (256), the Mathisson–Papapetrou equations are satisfied by Equations (259)–(261), so that the plain component of cause fits into GRT. If the plain component is regarded as a 1-component system (and not as a mixture component), the gravitation generating energy–momentum tensor is as expected according to Equations (257)${}_{2}$ and (258)

(262) $$\Theta_{P}^{ab}\;=\;T_{P}^{\left(ab\right)}.$$

The situation changes, if the plain component is regarded as a mixture component of a 2-component mixture whose second component is introduced in the next section.

#### 10.2.2. The Ghost Component

The balance equations defining the ghost component (G) are

(263) $$\begin{array}{ccc} N_{k}^{G} & = & \varrho^{G}u_{k}^{G}\;=\;0, \end{array}$$

(264) $$\begin{array}{ccc} T_{G}^{\left(ab\right)} & = & 0,\;T_{G}^{\left[ab\right]}{}_{;a}\;\overset{{}_{B}}{=}\;k_{G}^{b}, \end{array}$$

(265) $$\begin{array}{ccc} S_{G}^{kab}{}_{;k} & \overset{{}_{B}}{=} & \frac{1}{c^{2}}m_{G}^{ab},\;S_{G}^{(ab)k}{}_{;k;a}\;=\;0. \end{array}$$

By definition, the ghost component is characterized by vanishing mass density (no “normal” material ($\varrho^{G}=0$), therefore the name “ghost”). The Mathisson–Papapetrou equations demand

(266) $$\frac{1}{c^{2}}m_{G\;;a}^{ab}\;=\;2k_{G}^{b},$$

a dependence between force and angular momentum densities. If the ghost component is regarded as a 1-component system, the gravitation generating energy–momentum tensor is according to Equations (257)${}_{2}$ and (258)

(267) $$\Theta_{G}^{ab}\;=\;-S_{G}^{(ab)k}{}_{;k}.$$

Surprisingly, a “non-material” system such as the ghost component has a gravitational effect.

Plain and ghost components form a 2-component mixture, which is discussed in the next section.

#### 10.2.3. The Plain-Ghost Mixture

According to the settings in Equations (5), (42) and (78), we obtain for the mixture by taking Equations (259), (263), (260)${}_{1}$, (264)${}_{1}$, and (261)${}_{2}$ into account

(268) $$\begin{array}{ccc} N_{k} & = & N_{k}^{P}+N_{k}^{G}\;=\;\varrho^{P}u_{k}^{P}, \end{array}$$

(269) $$\begin{array}{ccc} T^{ab} & = & T_{P}^{\left(ab\right)}+T_{G}^{\left[ab\right]}\;⟶\;T^{\left(ab\right)}\;=\;T_{P}^{\left(ab\right)},\;T^{\left[ab\right]}\;=\;T_{G}^{\left[ab\right]}, \end{array}$$

(270) $$\begin{array}{cc} & \;⟶T^{ab}{}_{;a}\;=\;T_{G}^{\left[ab\right]}{}_{;a}, \end{array}$$

(271) $$\begin{array}{ccc} S^{abc} & = & S_{P}^{abc}+S_{G}^{abc}\;⟶\;S^{(ab)c}{}_{;c}\;=\;S_{G}^{(ab)c}{}_{;c}, \end{array}$$

(272) $$\begin{array}{cc} & \;⟶S^{kab}{}_{;k}\;=\;S_{P}^{kab}{}_{;k}+S_{G}^{kab}{}_{;k}\;=\;\frac{1}{c^{2}}m_{G}^{ab}. \end{array}$$

For fitting the GCCT plain-ghost mixture into the GRT, the Mathisson–Papapetrou equations (Equations (255) and (256)) have to be satisfied for the mixture. Taking Equations (261)${}_{1}$, (270) and (272) into account, Equation (255) results in

(273) $$\frac{1}{c^{2}}m_{G}^{ab}\;=\;2T_{G}^{\left[ab\right]}.$$

According to Equations (260)${}_{2}$ and (270)${}_{2}$, Equation (256) yields Equation (266).

The gravitation generating energy–momentum tensor of the plain-ghost mixture is according to Equations (257), (269)${}_{2}$ and (271)${}_{2}$

(274) $$\Theta^{ab}\;=\;T^{\left(ab\right)}-S^{(ab)c}{}_{;c}\;=\;T_{P}^{\left(ab\right)}-S_{G}^{(ab)c}{}_{;c}\;=\;\Theta_{P}^{ab}+\Theta_{G}^{ab}$$

which is different from Equations (262) and (267).

### 10.3. “Dark Matter” as a Ghost Component?

The previous statements allow discussing the following (strange) situation: An observer takes the plain-ghost mixture for a 1-component plain mixture because the ghost component is “dark”: no additional mass density and no spin can be detected from the point of view of the plain component. Erroneously, this observer supposes that Equations (259)–(261) are valid, but, in fact, Equations (268)–(272) are true. Observations of gravitational effects yield that the gravitation generating energy–momentum tensor $\Theta_{P}^{ab}$ in Equation (262) does not describe the observed gravitation because the ghost component of the plain-ghost mixture is invisible for the observer according to Equation (263). This situation remembers lively the search for “dark matter”, which should correct the energy–momentum tensor of the “visible matter”. If the observer speculates that the “dark matter” is a matter-free and spin-equipped object according to Equations (263)–(265), the gravitation generating energy–momentum tensor $\Theta^{ab}$ describes the gravitation correctly in contrast to $\Theta_{P}^{ab}$.

One question arises: Does a ghost component exist in nature and what is its physical essence? It is evident according to Equation (263) that the dark matter candidate “superheavy gravitino” [44] even though its mass may fit the plain-ghost mixture because of $\varrho^{G}\approx 0$, but the coupling in Equation (266) of the external quantities force and momentum densities remains mysterious.

## 11. Summary

A multi-component system is formed by its components, which are characterized by own individual quantities, such as velocity, density, chemical potential, stress tensor, temperature, heat flux, entropy flux densities, entropy production, supply and further items. All these individual quantities determine those of the multi-component system, which is described as a mixture. Individual temperatures of the components result in multi-temperature relaxation towards the corresponding equilibrium generating a common temperature of all components and of the mixture. A temperature of the mixture in multi-temperature relaxation non-equilibrium depends on the used thermometer and cannot be defined unequivocally.

Starting out with the rest mass densities of the components of the multi-component system, the mass flux densities of the components are defined by introducing their different 4-velocities. The mixture of the components is characterized by several settings. The first one is the additivity of the component’s mass flux densities to the mass flux density of the mixture. In combination with the mixture axiom, this setting allows defining mass density and 4-velocity of the mixture and the diffusion fluxes of the components. The non-symmetric energy–momentum tensor of one component interacting with the mixture is introduced, and its (3+1)-split together with the component’s mass and diffusion flux densities are generating the entropy identity [11], which restricts possible arbitrariness of defining.

The exploitation of the entropy identity requires additional settings, which result in physical interpretations of entropy density, flux and supply inducing the entropy production from the entropy identity. By use of the entropy identity, Lagrange multipliers are introduced concerning the constraints taken into account. These Lagrange multipliers are temperature, chemical potential and an additional non-equilibrium variable which characterizes the considered component to be a part of the mixture. Beside the classical irreversible processes—diffusion, chemical reactions, heat conduction and friction—an additional irreversible process—multi-temperature relaxation—appears due to the embedding of the considered component into the mixture. Different from the classical case, the mass production term, the heat flux density and the viscous tensor are modified by so-called effective quantities.

Equilibrium is defined by equilibrium conditions, which are divided into necessary and supplementary ones [11,12,45]. The necessary equilibrium conditions are given by vanishing entropy production, vanishing entropy flux density and vanishing entropy supply. Supplementary equilibrium conditions are: vanishing diffusion flux densities, vanishing component time derivatives (except that of the 4-velocity) and vanishing of the mass production terms. Presupposing these equilibrium conditions, we obtain: all components have the same 4-velocity, all heat flux densities are zero, the power as well as the divergence of the 4-velocity of each component vanish, and the viscous tensor is perpendicular to the velocity gradient.

The corresponding free component is defined by undistinguishable component indices (that is not the mixture which is a multi-component system). This 1-component system represents the easiest classical case, serving as a test of whether the interacting component in the mixture is correctly described. The vanishing of the entropy production in equilibrium is shortly investigated: the so-called Killing relation of the vector of 4-temperature is neither a necessary nor a sufficient condition for equilibrium. In addition, the statement that materials are perfect in equilibrium cannot be confirmed.

Finally, a special 2-component mixture is considered in the framework of General Relativity resulting in: a matter-free spin component influences the gravitation of the matter component. 

## Appendix A. Appendices

### Appendix A.1. Rest Mass Densities

Consider two frames, $B^{A}$ and $B^{B}$. $B^{A}$ is the rest frame of the ${}^{A}$-component and $B^{B}$ that of the ${}^{B}$-component. The corresponding rest mass densities are

(A1) $$\mathrm{rest}\;\mathrm{mass}/\mathrm{rest}\;\mathrm{volume}:\;\varrho^{A}\;:=\;\frac{m_{0}^{A}}{V_{0}^{A}},\;\varrho^{B}\;:=\;\frac{m_{0}^{B}}{V_{0}^{B}}.$$

It is not necessary, but for comparing the rest mass densities, we should choose the rest volumes to be equal

(A2) $$V_{0}^{A}\;≐\;V_{0}^{B}\;≐\ldots \ldots =:\;V_{0}.$$

By definition, the rest mass densities do not depend on the frame, which means the rest mass densities are *relativistic invariants* and should not be confused with the measured densities in non-resting frames

(A3) $$B^{B}:\;\varrho_{B}^{A}\;=\;\frac{\varrho^{A}}{1-v_{AB}^{2}/c^{2}},\;\;B^{A}:\;\varrho_{A}^{B}\;=\;\frac{\varrho^{B}}{1-v_{BA}^{2}/c^{2}},\;v_{AB}\;=\;-v_{BA}.$$

Here, $\varrho_{B}^{A}$ is the density of the ${}^{A}$-component in the rest frame of the ${}^{B}$-component, and $v_{AB}$ is the translational 3-velocity of $B^{A}$ in the frame $B^{B}$. These densities are out of scope in this paper.

We now consider Equation (9)${}_{1}$ in the rest frame $B_{O}$ of the mixture, which is defined by $u_{O}^{k}=\left(0,0,0,c\right)$. Consequently, we obtain

(A4) $$f_{O}^{A}\;=\;\frac{1}{c^{2}}u_{4O}^{A}c\;=\;\frac{1}{c}\frac{c}{\sqrt{1-v_{AO}^{2}/c^{2}}}.$$

Inserting Equation (A4) into Equation (9)${}_{2}$ results in the mass density of the mixture in its rest frame

(A5) $$B_{O}:\;\varrho_{O}\;=\;\sum_{A}f_{O}^{A}\varrho_{O}^{A}\;=\;\sum_{A}\frac{1}{\sqrt{1-v_{AO}^{2}/c^{2}}}\frac{\varrho^{A}}{1-v_{AO}^{2}/c^{2}}\;=\;\sum_{A}\frac{\varrho^{A}}{{\left(1-v_{AO}^{2}/c^{2}\right)}^{3/2}}.$$

The same result is obtained, if Equation (5)${}_{3}$ is written down for the rest system of the mixture.

### Appendix A.2. Example: Uniform Component Velocities

If there exists a common rest frame $B^{0}$ for all ${}^{A}$-components

(A6) $$u_{k}^{A}\;≐\;u_{k}^{0},\;\forall A.$$

According to Equation (5)${}_{3}$, we obtain

(A7) $$\varrho u_{k}\;=\;u_{k}^{0}\sum_{A}\varrho^{A}\;⟶\;\varrho c^{2}\;=\;u^{k}u_{k}^{0}\sum_{A}\varrho^{A}\;\wedge \;\varrho u_{k}u^{0k}\;=\;c^{2}\sum_{A}\varrho^{A},$$

and with Equation (9)${}_{1}$ follows

(A8) $$\varrho c^{2}\;=\;c^{2}f^{0}\sum_{A}\varrho^{A}\;\wedge \;\varrho c^{2}f^{0}\;=\;c^{2}\sum_{A}\varrho^{A}\;⟶\;{\left(f^{0}\right)}^{2}\;=\;1,$$

resulting in

(A9) $$f^{0}\;=\;\pm 1.$$

We obtain from Equation (9)${}_{2}$

(A10) $$\varrho \;=\;f^{0}\sum_{a}\varrho^{A}\;=\;\pm \sum_{a}\varrho^{A}\;⟶\;f^{0}\;=\;+1,$$

and taking Equation (A7)${}_{1}$ into account

(A11) $$u_{k}\;=\;u_{k}^{0}.$$

As expected, the 4-velocity of the mixture is identical with the uniform component velocities.

### Appendix A.3. Stoichiometric Equations

The system of the relativistic stoichiometric equations runs as follows

(A12) $$\begin{array}{c} \sum_{A}\nu_{\alpha }^{A}M_{0}^{A}\;=\;0,\;\\ \mathrm{component}\;\mathrm{index}:A=1,2,\ldots ,Z,\;\mathrm{reaction}\;\mathrm{index}:\alpha =1,2,\ldots ,\Omega . \end{array}$$

The stoichiometric coefficients $\nu_{\alpha }^{A}$ are scalars, and the partial rest mole mass $M_{0}^{A}$ is defined using the scalar mole number $n^{A}$ and the mole concentration $\zeta^{A}$ of the ${}^{A}$-component

(A13) $$M_{0}^{A}\;:=\;\frac{m_{0}^{A}}{n^{A}}\;=\;\frac{V_{0}}{n^{A}}\varrho^{A}\;=\;\frac{\varrho^{A}}{\zeta^{A}},\;\zeta^{A}\;:=\;\frac{n^{A}}{V_{0}}$$

according to Equations (A1) and (A2). The stoichiometric coefficients $\nu_{\alpha }^{A}$ are determined by the partial rest mole masses $M_{0}^{A},\;A=1,2,\ldots ,Z,$ before and after the αth reaction.

The time derivative of the mole number is determined by the reaction velocities ${\overset{•}{\xi }}_{\alpha }$

(A14) $$\overset{•}{n}{}^{A}\;=\;\sum_{\alpha }\nu_{\alpha }^{A}{\overset{•}{\xi }}_{\alpha }.$$

Multiplication with $M_{0}^{A}$ results by use of Equation (A12) in

(A15) $$M_{0}^{A}\overset{•}{n}{}^{A}\;=\;\sum_{\alpha }\nu_{\alpha }^{A}M_{0}^{A}{\overset{•}{\xi }}_{\alpha }\;⟶\;\sum_{A}M_{0}^{A}\overset{•}{n}{}^{A}\;=\;0.$$

Starting with the physical dimensions

(A16) $$\left[\nu_{\alpha }^{A}\right]\;=\;mol,\;\;\left[n\right]\;=\;mol,\;\;\left[M_{0}^{A}\right]\;=\;\frac{kg}{mol},\;\;\left[\zeta^{A}\right]\;=\;\frac{mol}{m^{3}},\;\;\left[{\overset{•}{\xi }}_{\alpha }\right]\;=\;\frac{1}{s},$$

that of the mass production term in the first row of Equation (141) is evidently

(A17) $$\left[{}^{\left(in\right)}\Gamma^{A}\right]\;=\;\frac{kg}{m^{3}s}.$$

A comparison with

(A18) $$\left[M_{0}^{A}\overset{•}{n}{}^{A}\right]\;=\;\frac{kg}{s}$$

shows that ${}^{\left(in\right)}\Gamma^{A}$ is the density which belongs to the mass production in Equation (A15). Because, according to Equation (A2), all rest mass densities are referred to the relativistic invariant $V_{0}$, we obtain from Equations (A18) and (A17) with Equation (A15)

(A19) $${}^{\left(in\right)}\Gamma^{A}\;=\;\frac{1}{V_{0}}\sum_{\alpha }\nu_{\alpha }^{A}M_{0}^{A}{\overset{•}{\xi }}_{\alpha }\;⟶\;\sum_{A}{}^{\left(in\right)}\Gamma^{A}\;=\;0.$$
